# Oxidative Stress Triggers Body-Wide Skipping of Multiple Exons of the Spinal Muscular Atrophy Gene

**DOI:** 10.1371/journal.pone.0154390

**Published:** 2016-04-25

**Authors:** Joonbae Seo, Natalia N. Singh, Eric W. Ottesen, Senthilkumar Sivanesan, Maria Shishimorova, Ravindra N. Singh

**Affiliations:** Department of Biomedical Sciences, Iowa State University, Ames, Iowa 50011, United States of America; Children's Hospital of Pittsburgh, University of Pittsburgh Medical Center, UNITED STATES

## Abstract

Humans carry two nearly identical copies of *Survival Motor Neuron* gene: *SMN1* and *SMN2*. Loss of *SMN1* leads to spinal muscular atrophy (SMA), the most frequent genetic cause of infant mortality. While *SMN2* cannot compensate for the loss of *SMN1* due to predominant skipping of exon 7, correction of *SMN2* exon 7 splicing holds the promise of a cure for SMA. Previously, we used cell-based models coupled with a multi-exon-skipping detection assay (MESDA) to demonstrate the vulnerability of *SMN2* exons to aberrant splicing under the conditions of oxidative stress (OS). Here we employ a transgenic mouse model and MESDA to examine the OS-induced splicing regulation of *SMN2* exons. We induced OS using paraquat that is known to trigger production of reactive oxygen species and cause mitochondrial dysfunction. We show an overwhelming co-skipping of *SMN2* exon 5 and exon 7 under OS in all tissues except testis. We also show that OS increases skipping of *SMN2* exon 3 in all tissues except testis. We uncover several new *SMN2* splice isoforms expressed at elevated levels under the conditions of OS. We analyze cis-elements and transacting factors to demonstrate the diversity of mechanisms for splicing misregulation under OS. Our results of proteome analysis reveal downregulation of hnRNP H as one of the potential consequences of OS in brain. Our findings suggest *SMN2* as a sensor of OS with implications to SMA and other diseases impacted by low levels of SMN protein.

## Introduction

Alternative pre-mRNA splicing plays an essential role in increasing the transcript diversity from a single gene [1]. All human genes with two or more exons are alternatively spliced in one or more tissues at one or more stages of development or distress [2]. Alternative splicing confers immense variability to the functional RNA motifs (sequence and structural) with implications to RNA-protein interactions that regulate and facilitate post-transcriptional regulation of gene expression, including RNA stability, RNA trafficking, mRNA translation, protein trapping and chromatin remodeling. Splicing is catalyzed by the spliceosome, a complex and dynamic machinery, comprised of hundreds of proteins that are differently assembled at different introns [3,4]. To add further complexity to the process, splicing is coupled with transcription, 5′-end capping and 3′-end polyadenylation of RNA [5]. A combinatorial control by cis-regulatory elements, RNA structure and transacting factors governs alternative splicing [6–9]. Mutations within regulatory sequences cause defective splicing and result in genetic diseases [10–12]. Genotoxic and oxidative stress (OS) conditions can also trigger aberrant splicing [13–15]. However, the mechanism of OS-induced aberrant splicing remains poorly understood.

Humans have two nearly identical *Survival Motor Neuron* (*SMN*) genes; *SMN1* and *SMN2* [16]. A full-length (FL) transcript and FL SMN protein are the major products of the *SMN1* gene, whereas the *SMN2* gene predominantly generates a truncated transcript and a truncated protein (SMNΔ7) due to skipping of exon 7 [17]. Unlike SMN, SMNΔ7 is only partially functional and highly unstable [18,19]. SMN plays a role in a variety of cellular processes, including transcription, snRNP biogenesis, DNA recombination, stress granule formation, vesicular transport, signal transduction, and motor neuron trafficking [20]. Deletion of and/or mutations in *SMN1* coupled with the inability of *SMN2* to compensate for the loss of *SMN1* leads to spinal muscular atrophy (SMA), a leading genetic disease of children and infants [21–23]. Mice contain a single *Smn* gene, which is equivalent to *SMN1*. Consistent with the indispensable nature of SMN, deletion of *Smn* gene leads to embryonic lethality [24]. However, introduction of *SMN2* into the null *Smn*^*-/-*^ background recapitulates a SMA phenotype [25]. While the role of *SMN2* remains elusive, it serves as a promising target for SMA therapy by compounds that elevate the levels of SMN by enhancing *SMN2* transcription and/or by correcting *SMN2* exon 7 splicing [20–22,26,27]. Since *SMN2* contributes towards the overall cellular pool of SMN, its copy number impacts the severity of SMA [25,28]. Evidence is emerging that SMN serves as a disease-modifying factor in other neurodegenerative disorders, including Parkinson’s disease and amyotrophic lateral sclerosis [29–31]. A recent study suggests a high level of SMN is required for the testicular development and male fertility [32].

OS is linked to several chronic conditions including cancer, cardiovascular diseases and neurodegenerative diseases [33]. Paraquat (PQ, 1,1′-dimethyl-4,4′-bipyridinium dichloride) is an environmental toxin and OS stimulant that is widely implicated in reactive oxygen species formation and mitochondrial dysfunctions [34–36]. PQ is commonly used in cell- and animal-based models to study the pathogenesis of Parkinson’s disease as well as to uncover the mechanism by which OS impacts various cellular processes [37–39]. However, the mechanism by which PQ causes splicing defects is poorly understood. A previous study has shown increased skipping of *SMN2* exons 5 and 7 in neuronal cells treated with PQ [40]. Employing a multi-exon-skipping detection assay (MESDA), we have recently demonstrated that splicing of multiple exons of *SMN1* and *SMN2* are affected by PQ-induced OS in both neuronal and non-neuronal cells [41]. Findings of these studies provide a clue that various *SMN* exons may be differently affected in different tissues subjected to PQ-induced OS. However, there is no in vivo study on how PQ affects tissue-specific splicing of various exons of *SMN* or any other gene harboring multiple skipping exons.

Here we examine the body-wide impact of PQ-induced OS on splicing of various *SMN2* exons in a healthy transgenic mouse model carrying the *SMN2* transgene. Our MESDA results reveal tissue-specific signatures of *SMN2* splice isoforms generated by OS. We analyzed cis-elements and transacting factors to uncover likely mechanisms by which splicing of various exons are impacted by OS.

## Materials and Methods

### Study approval

All experiments with mice and experimental guidelines were approved and monitored by the Institutional Animal Care and Use Committee (IACUC) at Iowa State University (Ames, IA, USA) following the federal and state guidelines. This study is designed to address how OS affects tissue-specific splicing regulation in mice exposed to PQ that is known to increase susceptibility to Parkinson’s disease.

### Animals

For all mice experiments, male and female mice were housed under the standard conditions of constant temperature (22 ± 1°C), humidity (relative, 30%) and a 12 h light/dark cycle. All mice had free access to food and water. Transgenic (TG) mice with FVB/N background, which have two copies of *SMN2* and one copy of *Smn* (*Smn*^+/-^;*SMN2*^+/+^), were generated from breeding pairs obtained from the Jackson Laboratory (JAX Strain 005024) [25]. Ear punches were used for genotyping.

### Design of in vivo experiments with paraquat

Each animal group treated with either phosphate buffered saline (PBS) (Life Technologies, Carlsbad, CA) or paraquat (PQ, 1,1′-dimethyl-4,4′-bipyridinium dichloride hydrate or methyl viologen dichloride hydrate, Sigma-Aldrich, St. Louis, MO) consisted of four 6–8 week-old TG mice: two male (~23 g each) and two female (~17 g each). PQ stock solution was prepared immediately before usage (200 mM in PBS). In PQ-treated group every individual mouse received a single injection (0.25 ml) of various amounts of PQ ranging from 10 to 70 mg/kg body weight using intraperitoneal (IP) route, similar to the dose range used in [42,43]. In PBS-treated control group, each mouse was given an IP injection of PBS (0.25 ml). Once injected, mice were monitored twice per day until their sacrifice. At the designated time points, mice were anaesthetized using isoflurane and sacrificed by cervical dislocation. Mouse tissues, including brain, heart, kidney, liver, lung, muscle, spinal cord, testis, and uterus/ovary, were collected and immediately frozen either in liquid nitrogen or on dry ice and moved to -80°C for storage until further usage.

### Survival study

Each 6–8 week-old TG mouse was randomly divided into two groups: control group (PBS injection, n = 10) and PQ group (70 mg/kg, n = 28). We chose the highest dose based on a previous study [43]. Mice were housed in groups of 3–4 same conditions as described above and provided with soaked standard laboratory food. Mice were monitored twice per day and euthanized if they showed signs of excessive pain or distress, including reduced motor activity. Unless otherwise stated, all experiments were terminated at 24 hours post PQ treatment. This termination time point was chosen based on the time course experiment that captured all splicing events affected by PQ-induced OS. At the termination point, mice were anaesthetized using isoflurane and sacrificed by cervical dislocation. All reasonable effort were made to minimize animal suffering, including anesthesia.

### Reverse transcription and MESDA

Total RNA was isolated from mouse tissues using TRIzol reagent (Life Technologies) following the manufacturer’s recommendations. RNA was subjected to DNase digestion using RQ1 RNase-free DNase (Promega, Madison, WI). DNase-treated RNA was recovered by either phenol:chloroform (OmniPur) extraction and ethanol precipitation or by using a Qiagen RNeasy Mini RNA purification kit (Qiagen, Venlo, Netherlands). RNA concentration was measured using a NanoDrop spectrophotometer (Thermo Scientific, Carlsbad, CA). cDNA was reverse transcribed from 1.6 μg of total RNA in a 10 μl reaction, using a SuperScript III Reverse Transcriptase (RTase, Life Technologies) and oligo(dT)_12-18_ primer (Life Technologies), random primers (Promega), or a gene-specific primer (3E8-Dde) for *SMN2* analysis. For mouse *Smn* analysis a gene-specific primer (3SmnE8) was used. One or two μl of RTase reaction were then used as a template for PCR amplification in a 25 μl reaction with Taq DNA Polymerase (New England Biolabs). The reaction was carried out in the presence of either a 5′-end-^32^P-labelled primer or a trace amount of [α-^32^P] dATP (3,000 Ci/mmole; Perkin-Elmer, Waltham, MA). MESDA was performed as previously described [41]. PCR products were resolved on native polyacrylamide gels. Analysis and quantifications of splice products were performed using a FPL-5000 Image Reader and Multi Gauge software (Fuji Photo Film Inc, Valhalla, NY).

Total RNA from cultured cells was prepared using TRIzol reagent (Life Technologies) following the manufacturer’s instructions. Reverse transcription reactions were carried by SuperScript III RTase. For amplification of *SMN* and *SBP2*, cDNAs were generated using gene-specific primers 3E8-Dde and 3hSBP2E4, respectively (S1 Table). For MESDA, cDNA was amplified using Taq DNA polymerase and a pair of primers 5SMNE1TSS and 3E8-25 (S1 Table), with the latter one being ^32^P-labeled at its 5′ end. 2 μl of RTase reaction were used per 25 μl of PCR reaction. RT-PCR amplified *SMN* splice products were resolved on native polyacrylamide gels. Analysis and quantifications were then performed using a FPL-5000 Image Reader and Multi Gauge software (Fuji Photo Film Inc). For *SBP2*, PCR amplification was done using primers 5hSBP2E1 and 3hSBP2E4 (S1 Table). RT-PCR amplified *SBP2* splice isoforms were resolved on native polyacrylamide gels. The gels were stained with ethidium bromides and the fluorescent images captured using the BioSpectrum AC Imaging System (UVP, Upland, CA). Primers used for RT-PCR and MESDA are given in S1 Table. Novel splice isoforms of *SMN2* are described in Table 1.

**Table 1:** Description of *SMN* splice variants.

Exon	Gene	GenBank accession number	Reference
ΔC3,7	*SMN2*	KF217140	This study
ΔC3,5,7	*SMN2*	KF217141	This study
ΔC5,6,7	*SMN2*	KF217142	This study
ΔC3–5,7	*SMN2*	KF217143	This study
ΔC3,5–7	*SMN2*	KF217144	This study
ΔC3–7	*SMN2*	KF217145	This study
ΔC5	*SMN1*	JQ657798	[41]
ΔC3	*SMN1*	JQ657800	[41]
ΔC5,7	*SMN1*	JQ657799	[41]
ΔC5,6,7	*SMN1*	JQ657801	[41]
ΔC5,6	*SMN1*	JQ732166	[41]
ΔC5,6	*SMN2*	JQ732167	[41]
ΔC5	*SMN1*	JQ657802	[41]
ΔC3	*SMN2*	JQ690861	[41]
ΔC3,4	*SMN1*	JQ745297	[41]
ΔC4,7	*SMN2*	JQ690864	[41]
ΔC3,7	*SMN2*	JQ690862	[41]
ΔC3,5	*SMN1*	JQ657803	[41]
ΔC3,5,7	*SMN2*	JQ690863	[41]
ΔC5,7	*SMN2*	JQ690865	[41]
ΔC3,7	*SMN1*	JQ690867	[41]
ΔC3,5	*SMN2*	JQ690866	[41]
ΔC3,5,7	*SMN1*	JQ690868	[41]

### Quantitative Real-time PCR (QPCR)

All reactions were performed in 96-well plates using the Stratagene Mx3005P PCR machine (Agilent technologies, Santa Clara, CA). cDNA was synthesized as described above using either oligo(dT)_12-18_ or random primers. Each RTase reaction was then diluted 1:20, and 1.5 μl was used in a 20 μl of PCR reaction containing 300 nM of each primer and either Brilliant II SYBR master Mix with Rox (Agilent Technologies) or FastStart Universal SYBR Green Master mix (ROX) (Roche, Basel, Switzerland). For a negative control, cDNA synthesized in absence of RTase or nuclease-free water was used as a template during QPCR amplification. The amplicon sizes were < 200 bp. Genes showing threshold cycle values greater than 38 were considered as undetectable/less expressed. To amplify different splice isoforms and to avoid amplification of genomic DNA, at least one primer in each primer set used for QPCR was designed to anneal to an exon-exon junctions. Each isoform abundance was calculated relative to the average of three samples from PBS-treated animals using the ΔΔCt method. Values were normalized by dividing by the geometric mean of the abundance of three genes: β-actin (ACTB), Glyceraldehyde-3-phosphate dehydrogenase (GAPDH), and Hydroxymethylbilane synthase (HMBS), amplified with primers described [44]. All samples were used in three biological replicates and amplified in technical duplicates.

### Western blot analysis

For animal tissues, frozen samples were thawed on ice and very briefly rinsed in ice-cold 1XPBS. Tissues were then homogenized in ice-cold Radioimmunoprecipation Assay (RIPA) buffer (Boston BioProducts, Ashland, MA) using the ratio of 100 μl of RIPA buffer per 10 mg of tissue. Immediately before usage, RIPA buffer was supplemented with either Halt protease single use inhibitor cocktail or Halt protease and phosphatase inhibitor cocktail (Thermo Scientific, Waltham, MA). Samples were then sonicated (6 times, 10 sec each, with 1 min intervals) and kept on ice for 30 min. The obtained lysates were subjected to centrifugation at 12,000 rpm for 30 min at 4°C to remove tissue debris. For cells grown in culture total protein lysates were prepared similar as described in [41]. Briefly, cell pellets were resuspended in ice-cold RIPA buffer (Boston BioProducts) supplemented with Halt protease and phosphatase inhibitor cocktail (Thermo Scientific). Resuspended cells were left on ice for 30 min, after what the lysates were spun down at 13,000 rpm for 10 min at 4°C to remove cell debris.

Protein concentrations were measured by Bradford protein assay using Bio-Rad protein assay solution (Bio-Rad, Hercules, CA). Protein samples were resolved on SDS-polyacrylamide gels. The separated proteins were transferred to Immobilon polyvinylidene fluoride (PVDF) membrane (Millipore, Billerica, MA) using the Turbo Transfer system (Bio-Rad). Membrane blocking was done in 5% nonfat dried milk in Tris-buffered saline with 0.05% Tween-20 (TBST). The following primary antibodies were used: mouse anti-SMN-KH antibody (1:500; Millipore), mouse monoclonal anti-SMN antibody (1:2,000, BD Transduction Laboratories, E. Ruthorford, NJ; cat # 610646), mouse anti-Gemin2 (1:500; Sigma-Aldrich; Clone 2E17, cat # G6669), rabbit polyclonal anti-elF4A2 (1:1000; Abcam, Cambridge, MA; ab31218), mouse monoclonal anti-SRPK2 (1:4000; BD Transduction Laboratories; cat # 611118), mouse monoclonal anti-phosphorylated SR proteins (1:200; Millipore; anti-phospho epitope SR proteins clone 1H4), rabbit monoclonal anti-PSF (1:10,000; Abcam; EPR11847, ab177149), goat polyclonal anti-p54 (1:3,000; Santa Cruz Biotechnology, Dallas, TX, C-16, sc-46220), rabbit polyclonal anti-SRp55 (1:200; Santa Cruz Biotechnology; H-180, sc-67100), mouse monoclonal anti-hnRNP A1 (1:10,000; Abcam; 9H19, ab5832), mouse monoclonal anti-hnRNP A2 (1:2,000; Abcam; DP3B3, ab6102), rabbit monoclonal anti-hnRNP K (1:10,000; Abcam; EP943Y, ab52600), mouse monoclonal anti-ASF/SF2 (1:400; US Biological Life Sciences, Salem, MA; S5555), goat polyclonal anti-TIA-1 (1:500, Santa Cruz Biotechnology; C-20, sc-1751), goat polyclonal anti-TIAR (1:200; Santa Cruz Biotechnology; C-18, sc-1749), goat polyclonal anti-hnRNP H (1:200; Santa Cruz Biotechnology; N-16, sc-10042), horseradish-peroxidase-conjugated anti-FLAG (1:4,000; Sigma-Aldrich; A8592), rabbit polyclonal anti-β-Actin (1:2,000; Sigma-Aldrich; A2066), mouse monoclonal anti-GAPDH (1:5,000; Abcam; 6C5, ab8245), rabbit monoclonal anti-Akt (1:1,000; Cell Signaling, Danvers, MA; C67E7, cat # 4691) and rabbit monoclonal anti-phospho-(Ser473)-Akt (1:1,000; Cell Signaling; D9E, cat # 4060). After incubation with primary antibodies, membranes were washed in TBST at least four times (10 min each) and incubated with the appropriate secondary antibody. The following secondary antibodies were used: horseradish-peroxidase-conjugated goat anti-mouse IgG (1:5,000; Jackson Immuno Research, West Grove, PA; cat # 115-035-003), donkey anti-rabbit IgG (1:2,000; GE Healthcare, Pittsburgh, PA; cat # NA934V) and donkey anti-goat IgG (1:2,000; Santa Cruz Biotechnology; sc-2020). Following multiple washes with TBST, membranes were developed using SuperSignal West Dura Extended Duration Substrate or SuperSignal West Femto Maximum Sensitivity Substrate (Thermo Scientific) following the manufacture’s instructions. Images were visualized using the BioSpectrum AC Imaging System (UVP). Often membranes were stripped using Restore Western Stripping Buffer (Thermo Scientific) and re-probed.

To detect 4-Hydroxynonenal adducts of histidine residues the membranes were incubated with primary mouse monoclonal anti-4-HNE antibody (R&D Systems, Minneapolis, MN; Clone #198960, MAB3249). Alexa Fluor 680-conjugated goat anti-mouse antibody (Thermo Scientific) was used as a secondary antibody. Western blot images were captured with a LI-COR Odyssey machine (LI-COR, Lincoln, NE).

For each Western blot, the mean intensity of each band was determined using ImageJ software. The mean intensity of the band of interest for each sample was divided by the mean intensity of β-actin, which served as a loading control. For comparisons purposes, the PBS average was set at 1.0 and PQ samples were expressed relative to this value.

### Sample preparation for isoelectric focusing (IEF)

TG brain tissues were homogenized in ReadyPrep Protein Extraction Kit (Bio-Rad) following the manufacturer’s instructions. Briefly, 100 mg of brain tissue was homogenized in the presence of 1 ml 2D Rehydration buffer containing protease inhibitors. This was followed by sonication (four times, 30 sec each with an interval of 30 sec). Lysates were clarified by centrifugation at 16,000 x g for 30 min at room temperature. The extracted proteins were cleaned up by acetone precipitation methods (TECH TIP#49, Thermo Scientific) and the concentration of proteins was measured via Bradford protein assay as described above.

### Two-dimensional polyacrylamide gel electrophoresis (2D-PAGE)

Isoelectric focusing (IEF) (pH3-10, 18 cm strip) using 500 μg of each sample was conducted in rehydration solution (8M Urea, 2% CHAPS, 0.5% immobilized pH gradients buffer, bromophenol blue containing 2.8 mg/ml DTT) at 20°C using the GE electrophoresis unit in accordance with the manufacturer’s instructions. After the IEF procedure, strips were incubated for 10 min with equilibration buffer (50 mM Tris-HCl, pH 8.8, 6M urea, 30% glycerol, and 2% SDS, bromophenol blue containing 10 mg/ml DTT). Equilibrated strips were then inserted onto SDS-PAGE gels (20 x 24 cm, 15%) and 2D gels were run. Upon run completion, the 2D gels were subjected to Coomassie Blue staining. Digital images from Coomassie stained gels were acquired using the Amersham Pharmacia Biotech ImageScanner flatbed scanner. Digitalized images were quantitatively analyzed using Progenesis SameSpots in accordance with the manufacturer’s instructions. Each spot was normalized by total valid spot intensity and the spots with a fold change greater than 2, an anova (p) less than 0.05 and a power greater than 0.8 were selected. Selected protein spots were enzymatically digested. Trypsin fragments were then analyzed using Matrix Assisted Laser Desorption/Ionization-Time of Flight (MALDI-TOF). Sequence tag searches were conducted using the MASCOT program.

### Cell culture

All the tissue culture supplies were from Life Technologies. Human HEK-293 cells were grown in Dulbecco’s Modified Eagle’s Medium (DMEM, cat # 11965) supplemented with 10% Fetal Bovine Serum (FBS). Human neuroblastoma SH-SY5Y cells were cultured in 1:1 mixture of Minimum Essential Medium (MEM, cat #11095) and F12 Medium (cat # 11765) supplemented with 10% FBS. Human primary fibroblasts from SMA type I patient (GM03813) obtained from Coriell Cell Repositories were cultured in MEM (cat # 10370) supplemented with 2 mM GlutaMAX-I (cat # 35050) and 15% FBS.

For PQ treatment of HEK-293, cells were pre-plated in 100 mm dishes at a density of ~1.9X10^6^ cells. For PQ treatment of SH-SY5Y and GM03813, cells were pre-plated in 60 mm culture dishes at a density of 4.2X10^6^ and 3.3X10^5^ per dish, respectively. Next day (24 hours later) medium in each dish was replaced with a fresh one without (-) or with (+) 1mM PQ. 24 hours later cells were washed with ice-cold 1XPBS (3 times) and collected by scraping. One sixth of the cells were used for total RNA preparation, while the rest of the cells were lysed using RIPA buffer.

HeLa cells were grown in DMEM (cat # 11965) supplemented with 10% FBS. To test whether different splice isoforms can be translated, HeLa cells pre-plated at a density of ~0.4x10^6^ cells per one well of a 6-well plate were transfected with 2 μg of 3XFLAG-tagged protein expression vector of interest using X-tremeGENE HP DNA transfection Reagent (Life Science) following the manufacturer’s recommendations. Twenty-four hours later, HeLa cells were washed with ice-cold 1X PBS (3 times) and collected by scraping for whole-cell lysate preparation using RIPA buffer similar as in [41].

### Generation of *SMN* splice isoform expression vectors

Human expression vectors for 3XFLAG-tagged SMN splice isoforms were generated as follows (S3 Table). cDNA of interest was reverse transcribed using total RNA from TG brain or SH-SY5Y cells, Super ScriptIII Reverse Transcriptase (Life Technologies) and either 3Adapter-oligo(dT) primer or a gene-specific primer (3E8-Dde). PCR amplification of each *SMN* isoform was performed with a pair of primers, 5SMNE1-MluI and P2-2 (S1 Table). The *SMN2* 3′UTR sequences were amplified using the primers 5SMNE8-St and 3Adapter-SalI. PCR products were gel-purified and used as templates together with the 3′UTR fragment for the second PCR with primers 5SMNE1-MluI and 3Adapter-SalI. PCR products were then digested with MluI and SalI and inserted into human 3XFLAG mammalian expression vector [45]. The identity of generated expression vectors was verified by sequencing.

### Motif analysis

Multiple EM for Motif Elicitation (MEME) package (version 4.11.1) [46] was used to identify over-represented motifs (4–6 width motif with more than 4 site counts) in exons with increased exclusion or inclusion upon PQ treatment. Analyses were performed with a search window of entire OS-induced aberrant splicing exons and 100 nt flanking intronic sequences.

### Statistical analysis

For Kaplan-Meier survival curves, log-rank tests were used. For QPCR, relative quantities with standard error were calculated by using ΔΔCT method in Excel (Microsoft Office 2011 edition). Statistical analyses were performed using the Student’s *t*-test. Unless otherwise mentioned, *P* values were two-tailed and the level of statistical significance was set at *P* < 0.05.

## Results

### Time and dose-dependent signatures of oxidative-stress-induced splice isoforms of *SMN2*

To analyze the impact of OS on splicing of *SMN2* pre-mRNA in vivo, we used a previously described transgenic (TG) mouse model that carries the human *SMN2* transgene on the *Smn*^*+/-*^ background [25]. Since the *Smn* gene is equivalent to human *SMN1*, this TG model is ideal for testing the impact of OS on *SMN2* splicing in the presence of normal levels of SMN protein. To induce OS in vivo and reliably capture the majority of splicing events at early time points, we treated TG mice with a single intraperitoneal (IP) injection of 70 mg/kg of PQ. This dose was selected based on a previous study [43]. Of note, barring few exceptions, most mice survived beyond 24 h of the PQ treatment (S1A Fig). Since we did not observe gender-specific differences in *SMN2* splicing outside the sex organs, males and females were analyzed together. Each treatment group was comprised of four animals: two males and two females. To confirm that OS conditions were induced in PQ treated mice, we monitored the level of 4-hydroxynonenal (4-HNE) adducts and phosphorylated AKT (pAKT) in brain samples. Both 4-HNE and pAKT are considered markers of OS [47,48]. Confirming the OS status in the brain, we observed an increase in the levels of 4-HNE and pAKT as early as 8 hours after PQ administration (S1B and S1C Fig).

**Fig 1:**
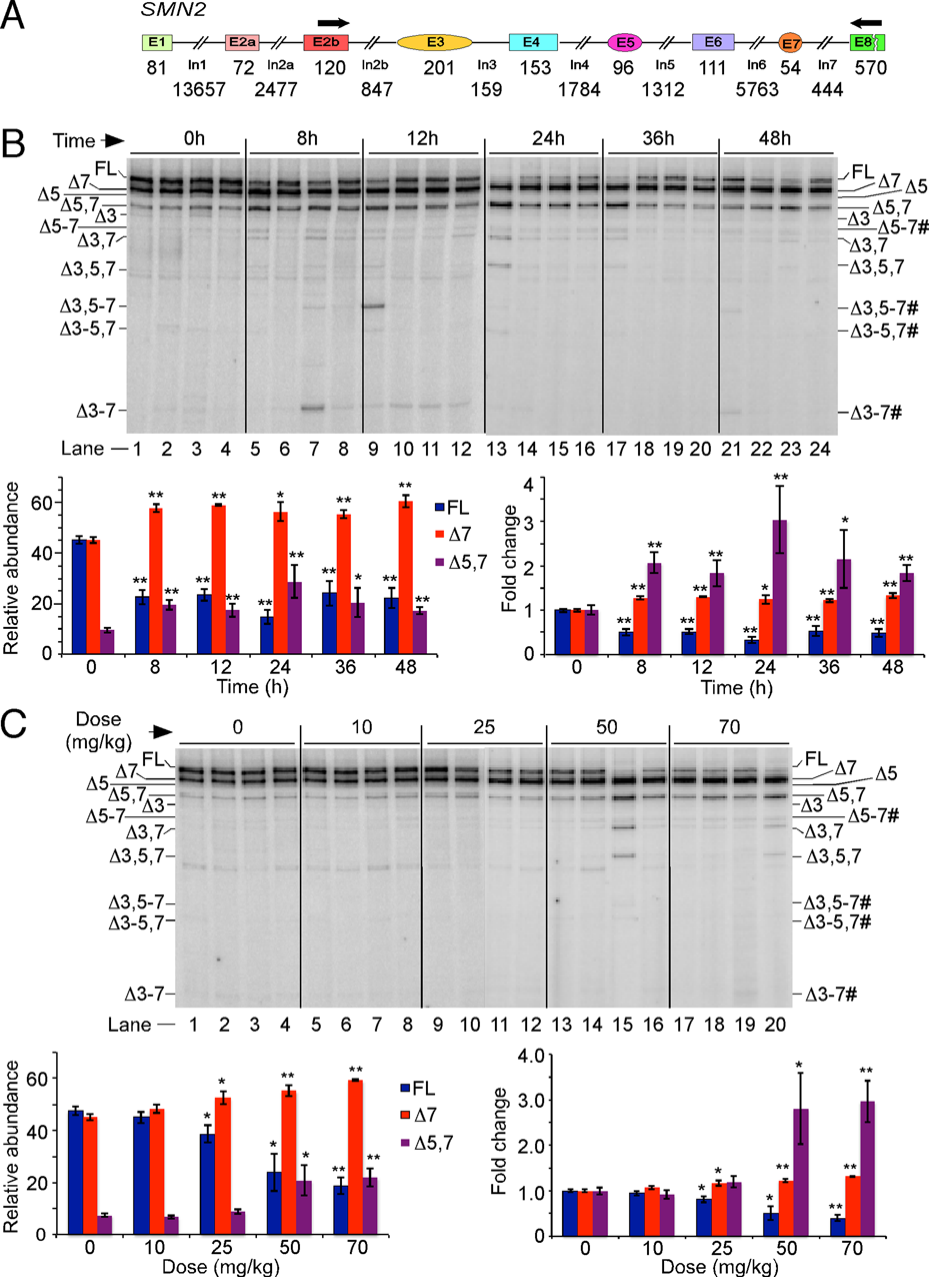
Time and dose dependent effects of PQ treatment on splicing of *SMN2* exons in the liver of TG mice. (A) Diagrammatic representation of the human *SMN2* gene. Exonic sequences are shown as boxes or ovals, whereas intronic sequences are shown as lines/broken lines. Annealing positions of primers used for MESDA are shown. Lengths of exons and introns are given in bp. Sizes of exons and introns are not to scale. (B) Detection of multiple exon skipping events of *SMN2* at different time points in liver. TG mice were IP injected with PQ (70 mg/kg) and liver was harvested at the indicated time points. For each time point, the first two lanes are males and the next two lanes are females. Splice products were analyzed by MESDA [41]. Splice variants are marked on the left and right sides of the gel. The identities of splice variants were established by sequencing. Pound signs next to splice variants mark novel isoforms (Table 1). Bar diagrams showing the relative abundance of individual transcripts as percent of total and the fold change are presented at the bottom of the gel. For fold change, the level of mRNA from the 0 h group was normalized to a value of 1. Error bars represent standard errors. Stars above PQ bars indicate statistical significance (*, *P <* 0.05; **, *P <* 0.01). Abbreviations: FL, full-length transcript; Δ, transcript lacking an exon(s); #, novel splice isoform; h, hour. (C) MESDA of *SMN2* at various PQ concentrations. Liver samples were harvested at 24 h post PQ injection. PQ concentrations are given at the top of the gel. At any given dose, the first two lanes are males and the next two lanes are females. Labeling and descriptions of bar diagrams are the same as in panel B.

To capture the earliest effect of OS on splicing of *SMN2*, we performed MESDA using liver samples harvested at different time points post PQ injection. Liver was chosen based on a consideration that it would have the fastest uptake of PQ delivered through IP route. To reduce the background, we used *SMN2*-specific primer to generate cDNA. As shown in Fig 1B, OS-induced changes in splicing of *SMN2* appeared as early as 8 h post PQ injection. In particular, we observed more than 50% decrease in the levels of the FL transcript, with a concomitant 2-fold increase in co-skipping of exons 5 and 7, while skipping of exon 7 increased by ~15% (Fig 1B). At 12 h post PQ treatment, the relative amount of splice isoforms remained similar to that of 8 h post PQ treatment (Fig 1B). However, at 24 h time point, there was a near total loss of the FL transcript accompanied by further increase in co-skipping of *SMN2* exons 5 and 7 (Fig 1B). At 36 h post PQ treatment, the levels of the FL transcript began to increase with simultaneous reduction in the levels of Δ5,7 transcripts; however, transcript levels were not fully restored even 48 h after PQ injection (Fig 1B). These results underscore that co-skipping of *SMN2* exons 5 and 7 is a signature event of PQ-induced OS observed during the first 24 h of treatment. Interestingly, 70 mg/kg treatment resulted in noticeably higher levels of Δ3–7 and Δ3–5,7 transcripts 8 and 12 h post injection, respectively, in two out of 20 samples (Fig 1B, lanes 7 and 9). We also detected several faint bands corresponding to splice isoforms in which multiple exons were simultaneously skipped; most of these isoforms lacked exon 3 and/or exon 5 (Fig 1B). Our results also revealed novel splice isoforms generated during the conditions of OS, such as Δ5–7, Δ3,5–7, Δ3–5,7 and Δ3–7 (Fig 1B, marked with pound signs). GenBank accession numbers of various *SMN* splice isoforms generated under normal and OS conditions are given in Table 1.

Based on our results described above we chose 24 h post PQ injection as the time point to collect samples for analysis of all aberrantly spliced *SMN2* variants. Of note, we did not take later time points to avoid the impact of secondary effects on splicing. The effect of PQ-induced OS on splicing of various *SMN2* exons was minimal, marginal and substantial at low (10 mg/kg), medium (25 mg/kg) and high (50 mg/kg) doses of PQ, respectively (Fig 1C). At 50 mg/kg treatment, we detected greatly increased levels of Δ5,7, Δ3,7 and Δ3,5,7 splice variants in liver of one out of four animals (Fig 1C, lane 15). These findings underscore individual variations in susceptibility of *SMN2* exons to skipping under OS. Overall, our results confirmed that co-skipping of exons 5 and 7 is the hallmark of *SMN2* aberrant splicing under the severe conditions of OS (50 and 70 mg/kg PQ treatment). However, under milder OS conditions induced by lower PQ (25mg/kg), increased skipping of *SMN2* exon 7 was the major event (Fig 1C).

### Tissue-specific effect of oxidative stress on splicing of various exons of *SMN2*

**Fig 2:**
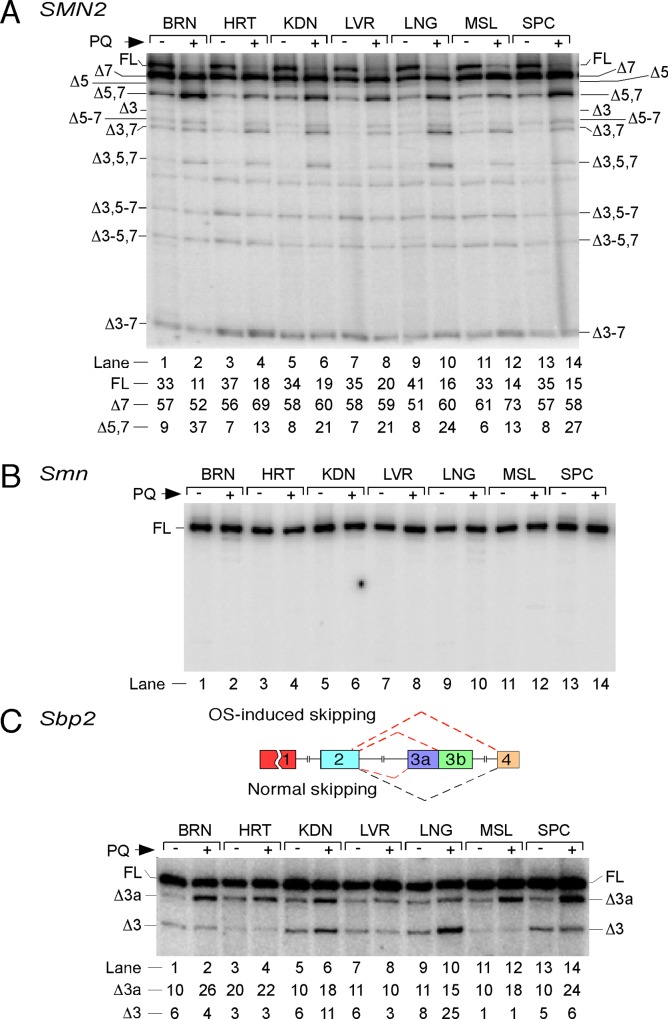
Effect of PQ treatment on splicing of *SMN2*, *Smn* and *Sbp2* in various tissues of a representative TG mouse. (A) MESDA of *SMN2* in different tissues harvested from a TG mouse treated with PBS or PQ (70 mg/kg). Samples were harvested at 24 h post PQ injection. Types of tissues are indicated on the top of the gel. Labeling and other descriptions are the same as in Fig 1B. Percentages of major splice isoforms are given at the bottom of the gel. They were calculated from the total value of FL, exon 7-skipped and exon 5,7-skipped products. Abbreviations: BRN, brain; HRT, heart; KDN, kidney; LVR, liver; LNG, lung; MSL, muscle; SPC, spinal cord. (B) MESDA of *Smn* in different tissues harvested from the same mouse as in panel A. No skipping of *Smn* exons was detected. Abbreviations are the same as in panel A. (C) Splicing pattern of *Sbp2* exon 3/3a in different tissues harvested from the same mouse as in panel A. Percentages of skipping of exon 3 and 3a are indicated at the bottom of the gel. They were calculated from the total value of FL, exon 3a-skipped and exon 3-skipped products. Diagrammatic representation of *Sbp2* alternative splicing under normal and OS conditions is given in the top panel.

We next examined the tissue-specific effect of OS on splicing of *SMN2* exons. We performed this analysis on various tissues collected 24 h post PQ injection (70 mg/kg; IP route). A representative example of MESDA is shown in Fig 2A. As expected, the Δ7 transcript was the major splice isoform in brain, heart, kidney, liver, lung, muscle and spinal cord of untreated animals, followed by the FL transcript (Fig 2A). The relative amount of Δ5,7 variant was a distant third in all untreated samples. However, we observed a near total disappearance of the FL transcript accompanied by an increase in the levels of Δ5,7 transcript in all organs and tissues harvested from PQ-treated mice (Fig 2A). Co-skipping of exon 5 and 7 was highest in brain and spinal cord (4 and 3.5 times increase, respectively) and lowest in heart and muscle (~ 2 times increase) (Fig 2A). Interestingly, heart and muscle were the only two organs in which PQ treatment resulted in a more than 10% increase in individual skipping of exon 7 (Fig 2A, Δ7 transcript). The levels of individual skipping of exon 5 and exon 3 were extremely low across all samples tested. Visual inspection of the gel indicated that PQ treatment decreased individual skipping of exon 5 and exon 3 (Fig 2A). With respect to the shorter splice variants, the levels of Δ3,7 and Δ3,5,7 transcripts were affected the most, particularly in lung (Fig 2A). However, these transcripts represented only a very small fraction of *SMN2* splice isoforms. As for the novel splicing events, the conditions of OS led to a small but noticeable increase in co-skipping of exons 5, 6 and 7, particularly in spinal cord (Fig 2A). At the same time, the levels of other novel splice variants, such as Δ3–7, Δ3,5–7 and Δ3–5,7 appeared to be largely unaffected by OS. When MESDA was performed with primers specific to mouse *Smn*, no effect of OS on splicing of any of the mouse *Smn* exons was observed in any of the tissues tested (Fig 2B).

To independently validate the results of MESDA, we analyzed transcripts by PCR employing primers that annealed to flanking exonic sequences of each individual alternatively spliced exon. Here again, we used *SMN2*-specific primers to generate cDNA. We first examined the splicing pattern of *SMN2* exon 7 that has the highest propensity of skipping under normal and OS conditions (Fig 2A, lanes 1–14). Confirming the results of MESDA, we observed overwhelming skipping of *SMN2* exon 7 in tissues harvested from the PQ-treated animals (S2B Fig). We next determined the splicing pattern of *SMN2* exon 5 by PCR using forward and reverse primers that annealed to exons 4 and 6, respectively. Consistent with the results of MESDA, PQ-induced OS caused small but noticeable skipping of *SMN2* exon 5 in all tissues tested (S2C Fig). Finally, we examined splicing of *SMN2* exons 2a, 2b and 3 by PCR employing forward and reverse primers that annealed to exons 1 and 4, respectively. PQ-induced OS produced small but noticeable skipping of *SMN2* exon 3 in all tissues examined (S2D Fig). Once again, these findings were consistent with the results of MESDA. Parallel experiments on *Smn* did not show any noticeable effect of OS on splicing of exons 3, 5 and 7 in any tissue examined (S2B–S2D Fig).

While MESDA revealed susceptibility of various *SMN2* exons to skipping under OS, we did not identify an OS-induced usage of an alternative splice site (ss) within any of the *SMN2* exons. We have previously shown the usage of an alternative 3′ ss within exon 3 of *Secis-Binding Protein 2* (*SBP2*) transcripts [49]. *SBP2* is a housekeeping gene responsible for the synthesis of selenoproteins in higher organisms [50]. SBP2 is also implicated in prolonging cell survival under OS [51]. We used *Sbp2* as a model to examine the OS-induced usage of the alternative 3′ ss in various tissues of TG mice. Of note, usage of an alternative 3′ ss within exon 3 causes skipping of exon 3a (the 5′ portion of exon 3), leading to the generation of a SBP2 isoform targeted to the mitochondria (Fig 2C) [49]. Our results show that PQ treatment enhanced skipping of *Sbp2* exon 3a in brain, kidney, muscle and spinal cord of TG mice (Fig 2C). We also observed the highest increase in skipping of exon 3 in lung of PQ-treated TG mice (Fig 2C). The kidney emerged as a unique tissue in which PQ enhanced skipping of both exons 3 and 3a (Fig 2C). Interestingly, OS did not affect either of these exons in liver, suggesting that factors responsible for the skipping of exons 3 or 3a are absent in this tissue (Fig 2C). Skipping of exon 3a in brain and several other tissues is possible only when the 3′ ss of exon 3b is strengthened with respect to the 3′ ss of exon 3. While intronic and exonic sequences at the 3′ ss of exon 3 are represented by U- and A-rich motifs, respectively, intronic and exonic sequences at the 3′ ss of exon 3b are represented by C-rich motifs (S3A Fig). Both exons 3 and 3b of *Sbp2* share the same 5′ ss that forms extensive base pairing with U1 snRNA, a component of U1 snRNP (S3B Fig); however, the 5′ ss of exons 3/3b is predicted to be sequestered in a stem-loop structure that may in part account for an OS-induced skipping of *Sbp2* exon 3 in kidney and lung (S3C Fig). In contrast to the skipping of *SMN2* exon 7, none of the exons of *Sbp2* showed universal skipping under OS. Of note, there are noticeable differences between mouse and human *SBP2* exon 3 and flanking intronic sequences (S3A Fig). Consequently, we did not observe any PQ-induced skipping of *SBP2* exon 3a in human neuronal SH-SY5Y cells (S3D Fig). We also determined splicing of *SBP2* exons 3/3a in cultured HEK-293 cells treated with PQ. Considering HEK-239 cells are derived from human embryonic kidney, the splicing pattern of *SBP2* exons 3/3a was consistent with splicing of mouse *Sbp2* exons 3/3a in kidney (compare S3D Fig with Fig 2C). These results suggest that motifs responsible for the kidney-specific splicing regulation of *SBP2* exons 3/3a are conserved between human and mice.

### Oxidative stress alters the cumulative frequency of several splicing events

**Fig 3:**
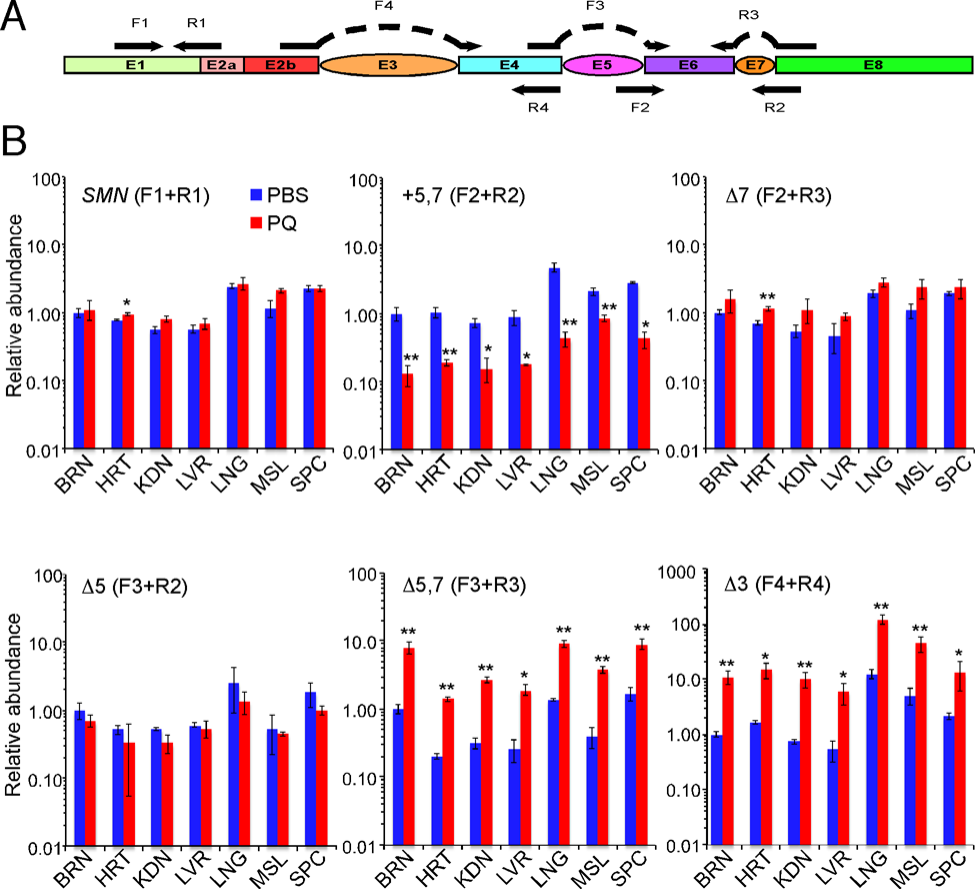
QPCR for specific splicing events of *SMN2* in different tissues of TG mice. (A) Diagrammatic representation of *SMN2* transcript. Ovals indicate alternatively spliced exons of *SMN2*. Annealing positions and names of primers used for QPCR are shown. (B) Frequency of specific splicing events of *SMN2* in different tissues. PQ treatment and tissue collection were the same as described in Fig 2A. QPCR products are indicated with names of skipped (Δ) or included (+) exons. Primer combinations used for QPCR are given in parenthesis. Expression levels relative to PBS treated brain for each isoform were normalized to value of 1. Error bars represent standard error. Stars above PQ bars indicate statistical significance (*, *P <* 0.05; **, *P <* 0.01). Abbreviations are the same as in Fig 2A.

To analyze the cumulative frequency of skipping of a specific *SMN2* exon, we employed quantitative real-time PCR (QPCR). To determine the levels of total *SMN2* transcripts, we used forward and reverse primer that annealed to exon 1 and the exon 1/exon 2a junction, respectively (Fig 3A). We chose this primer combination based on the fact that neither exon 1 nor exon 2a is alternatively spliced (S2D Fig). To evaluate one- or two-exon skipping events, we used primer sets in which at least one primer annealed to an exon-exon junction (Fig 3A). Of note, using a wild type mouse as a control, we confirmed that our primer combinations do not amplify *Smn* transcripts. Final values were normalized against the geometric mean of three non-fluctuating controls in the same tissue [44]. The results were expressed in terms of relative abundance of transcripts in normal and OS conditions (Fig 3B). Since oligo(dT)_12-18_ was used to prepare cDNA, all transcripts analyzed in this study were polyadenylated. Our results revealed that PQ-induced OS does not alter the overall levels of *SMN2* transcripts in any tissue except heart, where a small but statistically significant increase was observed (Fig 3B). Considering data were normalized against the internal controls, our results of QPCR should be interpreted with caution. For instance, a general decline in the rate of transcription due to an energy deficit created by OS will not be reflected.

Consistent with the results of MESDA (Fig 2A), we observed a substantial decline in the levels of FL transcript (+5,7 variant) with a concomitant increase in the levels of Δ5,7 splice variant in all examined tissues of PQ-treated animals (Fig 3B). At the same time, the levels of Δ7 transcript were not affected by the conditions of OS in any samples except for heart. In this organ, skipping of exon 7 increased slightly but significantly (Fig 3B). Further, individual skipping of exon 5 showed a trend of decreasing under OS, although this decrease was not statistically significant (Fig 3B). Of note, Ct value for amplification of this transcript was highest (between 34 and 37) among all splice variants whose levels we tested by QPCR. The results of QPCR also revealed that OS induced by PQ promoted skipping of *SMN2* exon 3 (Fig 3B). Note that because of the primer design, transcripts with individually skipped exon 3 as well as transcripts with co-skipped exons 3, 5 and 7 will also be amplified in this QPCR. As per MESDA results, the levels of the latter transcripts were increased in PQ-treated animals (Fig 2A). Due to technical limitations, we were unable to validate the levels of specific transcripts (such as Δ3,7 and Δ3,5,7) by QPCR. We also used brain samples to quantify intron-retained products. Except for a reduced rate of retention of *SMN2* intron 1, our results of QPCR revealed no significant difference in intron retention between normal and PQ-treated samples (S4 Fig). Thus, enhanced skipping of exons, individual or multiple, appears to be the major consequence of OS for *SMN2* splicing.

### Oxidative stress differentially affects splicing of various *SMN2* exons in reproductive organs

**Fig 4:**
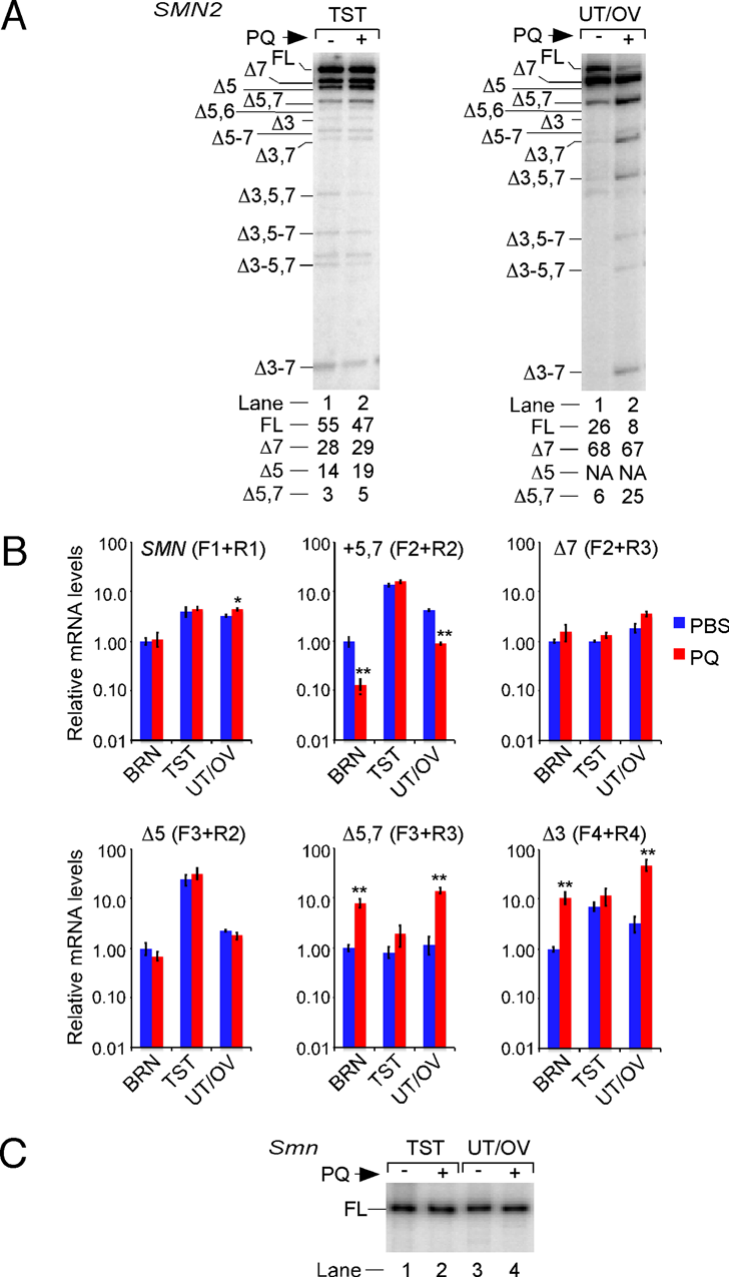
Effect of PQ treatment on splicing of *SMN2* and *Smn* in reproductive organs of TG mice. (A) MESDA of *SMN2* in testis and uterus/ovary harvested from a TG mouse treated with PBS or PQ (70 mg/kg). Samples were harvested at 24 h post PQ treatment. Labeling and other details are the same as in Fig 2A. Percentages of major splice isoforms are indicated at the bottom of the gel. Abbreviations: BRN, brain; TST, testis; UT/OV, uterus/ovary. (B) Comparison of splice variants of *SMN2* in the indicated tissues of TG mice determined by QPCR. The descriptions of QPCR are the same as in Fig 3. Abbreviations are the same as in panel A. (C) MESDA of *Smn* in testis and uterus/ovary harvested from the same animal as in panel A.

We also examined the relative abundance of various *SMN2* transcripts in mouse reproductive organs harvested at 24 h post PQ treatment. Consistent with previous reports in two different mouse models of SMA [32,52], we observed FL *SMN2* transcript as the major splice isoform in testis of untreated animals. Similar to a recent report employing a mild mouse model of SMA [32], testis of untreated TG mice showed noticeably higher levels of *SMN2* exon 5 skipping compared to other organs (Fig 4). We also detected low levels of co-skipping of *SMN2* exons 5 and 6 (Fig 4A). Interestingly, PQ treatment did not produce significant changes in the splicing of any of the *SMN2* exons (Fig 4A). We observed only a small decrease in the levels of the FL transcripts (Fig 4A). The relative levels of various *SMN2* transcripts in uterus/ovary with or without PQ treatment were almost identical to those observed in brain or spinal cord (compare Figs 4A and 2A).

We also performed QPCR to determine the frequency of specific splicing events in PQ-treated reproductive organs. As a control we used brain from the same animal. Validating the results of MESDA (Fig 4A), we observed no appreciable change in the splicing pattern of any of the *SMN2* exons in testis of TG mice injected with PQ (Fig 4B). Similar to brain, enhanced skipping of *SMN2* exons 3, 5 and 7 were major events in uterus/ovary of TG mice treated with PQ (Fig 4B). Also, most of the OS-induced skipping of *SMN2* exon 5 in uterus/ovary occurred in transcripts that lacked exon 7.

### Suboptimal splice sites contribute towards oxidative-stress-induced aberrant splicing of exons

**Fig 5:**
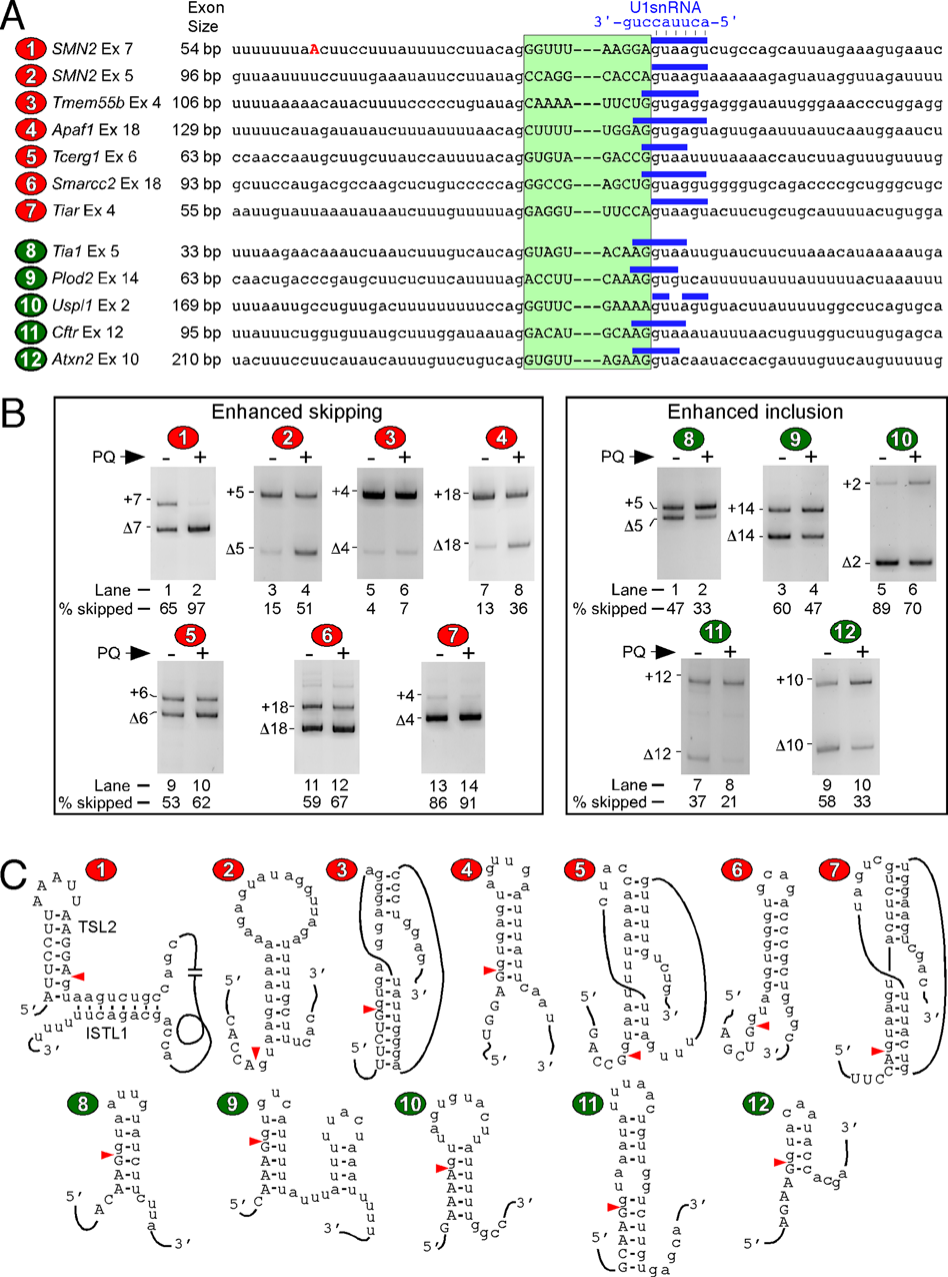
Analyses of splice sites of OS-sensitive exons of *SMN2* and other murine genes. (A) Names of exons and their sizes are indicated. Nucleotides involved in the base paring between the 5′ ss of a given exon and U1 snRNA are marked by solid horizontal bars on the top of each sequence. Exonic sequences are indicated by uppercase letters, while introns are indicated by lowercase letters. Skipping of exons numbered 1–7 within red ovals is increased, while skipping of exons numbered 8–12 within green ovals is decreased upon PQ treatment. (B) Splicing pattern of OS-sensitive exons in the presence of PBS or PQ treatment. PQ treatment and tissue collections were same as described in Fig 2A. (C) Manually predicted secondary structures of the 5′ ss of OS-sensitive exons. Arrowheads indicate the 5′ ss.

To assess the potential contribution of cis-regulatory elements and splice sites to OS-induced aberrant splicing, we compared sequences of seven exons whose skipping was increased by OS (Fig 5A and 5B). We also analyzed their flanking intronic sequences. Except for *SMN2* exon 5 and 7, other exons were chosen randomly. The OS-induced changes in the splicing pattern of these exons were assessed by RT-PCR, using cDNAs from brain generated with oligo(dT)_12-18_. For the PCR step, we used primers that annealed to sequences of exons that flank an OS-sensitive exon of interest. Usually exons that are included have G residues at their first and last positions [53,54]. Underscoring the weak 3′ and/or 5′ ss as the possible contributor to exon skipping under the conditions of OS, five of the seven skipped exons lacked the preferred R (purine) and/or G residues at the first and/or the last positions of exons, respectively. Exceptions to this rule were exon 6 of *Tcerg1* and exon 18 of *Smarcc2*. The splice sites of these exons might be suboptimal due to other reasons. For instance, the presence of a U residue at the 5^th^ position of *Tcerg1* intron 6 shortens the region base-paired with U1 snRNA, thus, hampering an efficient recruitment of the U1 snRNP to the 5′ ss of *Tcerg1* exon 6 (Fig 5A).

We also analyzed splice sites of five alternatively spliced exons that we found to be included in response to OS caused by PQ (Fig 5A and 5B). All of these candidate exons had a favorable R residue at the first exonic position. Four of these exons also had a favorable G residue at the last exonic position. Under normal conditions, three of the five exons were predominantly skipped (Fig 5B; *Plod2* exon 14, *Uspl1* exon 2 and *Atxn2* exon 10). All of these exons appear to have suboptimal 5′ ss due to poor base pairing between U1 snRNA and the 5′ ss (Fig 5A).

Several studies have implicated the role of RNA structure in regulation of *SMN2* exon 7 splicing [55–57]. In particular, we have demonstrated that the 5′ ss of *SMN* exon 7 is sequestered by a terminal stem-loop structure (TSL2), as well as by an internal stem formed by the long-distance interaction (ISTL1) [56,57]. We also analyzed the structural context of other eleven OS-sensitive exons described in Fig 5A and 5B. For most exons, the predicted structure showed a strong terminal or internal stem encompassing the 5′ ss or its vicinity (Fig 5C). However, we did not observe any structure(s) specific for exon exclusion versus inclusion triggered by OS.

We then analyzed what motifs were enriched within these twelve exons and their flanking intronic sequences. Two hexameric motifs (MAYCAM and AKYMAR), a purine-rich motif (ARAGA) and a GC-rich motif (CTGKRY) were present respectively in seven, six and five exons skipped under OS (S5A Fig). In addition, two (AKWGST and ACAG) and four motifs (CCCT, GYRRGT, GCTTWC and WGWGRA) were enriched in upstream and downstream introns of most of these skipped exons (S5A Fig). At the same time, four motifs (ACWMRG, GBTCA, GWAR and CCAAVT) were enriched in at least four of the five exons that showed increased inclusion in response to OS (S5B Fig). In addition, two (MASAMA and TSCMTS) and three motifs (SCWC, CAWAM and ACTA) were present respectively in upstream and downstream introns of these exons (S5B Fig). The variety of the above-listed enriched motifs suggests involvement of multiple protein factors in splicing regulation of these exons under normal and OS conditions.

### Effect of oxidative stress on levels of SMN protein and regulatory factors in TG brain

**Fig 6:**
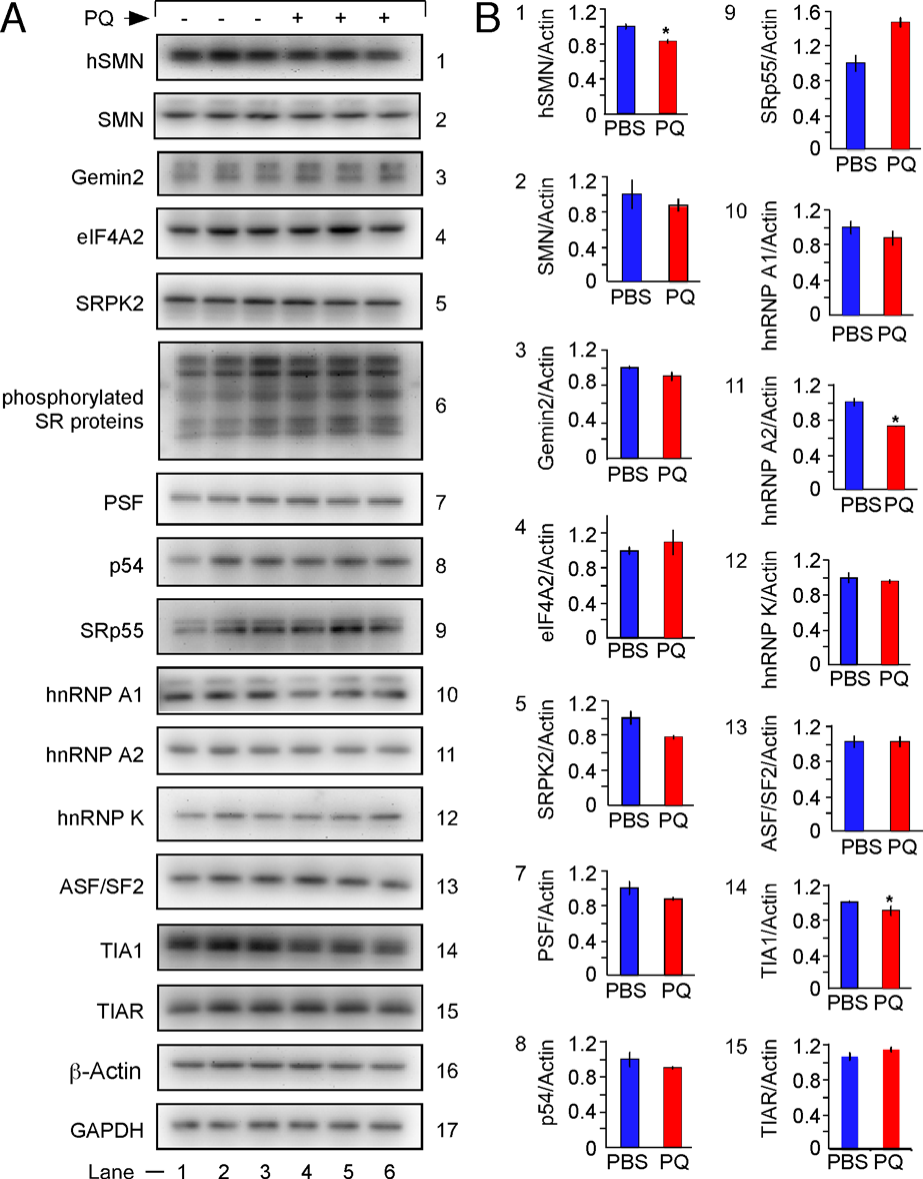
Effect of PQ treatment on the levels of various proteins in the brain of TG mice. PQ treatment and tissue collections were the same as described in Fig 2A. (A) Western blots showing the expression levels of SMN, and the indicated proteins in PBS or PQ-treated TG brain. PQ treatment and brain collection were the same as described in Fig 2A. Depending on the protein of interest 10, 20 or 30 μg of total lysate was loaded per lane. The same membrane was stripped and re-probed for evaluation of up to four target proteins. Equal protein loading was confirmed by re-probing the blot with antibodies against β-actin and GAPDH. Names of the probed proteins are indicated on the left of the blot. (B) Densitometric analysis of Western blots shown in panel A. Stars above bars indicate statistical significance (*, *P* < 0.05).

To interrogate the role of factors that affect splicing and post-splicing events of *SMN2* under the conditions of OS in vivo, we examined the levels of several proteins in TG brain within the first 24 h of PQ treatment. We began with the determination of SMN levels, since an OS-induced decline in SMN levels will have a direct impact on snRNP assembly and genome-wide perturbations of splicing. Both *Smn* and *SMN2* contribute to the overall SMN pool in TG mice. Hence, we used two different antibodies to distinguish human SMN (hSMN) from total SMN (hSMN + mSmn). Because of the overwhelming skipping of *SMN2* exons 5 and 7 induced by OS (Figs 2A, 3B and 4B), we expected to see a decline in the levels of hSMN in brain of TG animals injected with PQ. We observed only a small but statistically significant reduction in hSMN in TG brain subjected to OS (Fig 6). A small decline in hSMN did not significantly change the total levels of SMN in TG brain subjected to OS (Fig 6). Of note, we confirmed that all *SMN* splice variants are capable of being translated when expressed ectopically in HeLa cells (S6 Fig). However, we did not detect any hSMN isoforms including those corresponding to *SMN2*ΔC7 and *SMN2*ΔC5,7 transcripts, the major splice isoforms observed under OS conditions. Skipping of *SMN2* exon 7 results in the generation of a protein degradation signal at the C-terminus of SMN [19]. Since skipping of exon 7 occurred in all prominent splice isoforms generated under OS, the degradation signal was the likely cause why we were unable to detect these proteins.

Gemin2 tightly interacts with SMN and contributes towards most (if not all) SMN-associated functions [20]. We did not observe a significant change in Gemin2, suggesting that the SMN-Gemin2 complex is not a limiting factor during the first 24 h of PQ-induced OS (Fig 6). Eukaryotic initiation factor eIF4A2 is involved in microRNA-mediated translational repression [58]. PQ-induced OS did not alter eIF4A2 level (Fig 6), suggesting that the general microRNA-mediated translation suppression is unlikely to be triggered during the initial stages of OS. Consistent with the insignificant change in the levels of serine/arginine (SR)-phosphorylating protein SRPK in TG brain subjected to OS, we did not observe a general change in the phosphorylation status of splicing factors (Fig 6). Nonetheless, these results should be treated with caution because enhanced phosphorylation of one protein could be masked by dephosphorylation of another protein of similar size. Further, SF2/ASF, an SR protein, has been previously implicated in strengthening of the 3′ ss of *SMN* exon 7 [59]. The strength of a 3′ ss could also be altered by hnRNP K, a critical splicing factor with important role in neuronal differentiation [60]. However, we did not observe any significant OS-induced changes in levels of these proteins in TG brain (Fig 6).

Splicing factors hnRNP A1 and hnRNP A2 are known negative regulators of *SMN2* exon 7 splicing [61,62]. These two proteins interact with both exon 7 and intron 7 of *SMN2* [63]. While levels of hnRNP A1 in TG brain subjected to OS remained unaffected, we observed a small but statistically significant decrease in the level of hnRNP A2 (Fig 6). These results suggest that the rules of exon skipping under conditions of OS might be governed by factors that are not necessarily negative regulators. Indeed, we observed a small but noticeable decrease in the level of TIA1, a glutamine-rich protein that serves as a key regulator of stress granule formation. We have previously reported TIA1 as a positive regulator of *SMN2* exon 7 splicing [45]. Similar to TIA1, PSF is another glutamine-rich protein that stimulates *SMN2* exon 7 inclusion. Based on sequence homology, PSF is grouped along with p54nrb/NONO proteins [64]. However, unlike TIA1, we did not find a statistically significant change in the levels of PSF and p54nrb proteins in TG brain upon PQ treatment (Fig 6). Level of another splicing factor, SRp55, that is known to interact with the 5′ ss of an exon [65], showed a noticeable but statistically insignificant increase in PQ-treated brain (Fig 6).

### Proteome analysis reveals a role for hnRNP H in OS-induced downregulation of SMN

**Fig 7:**
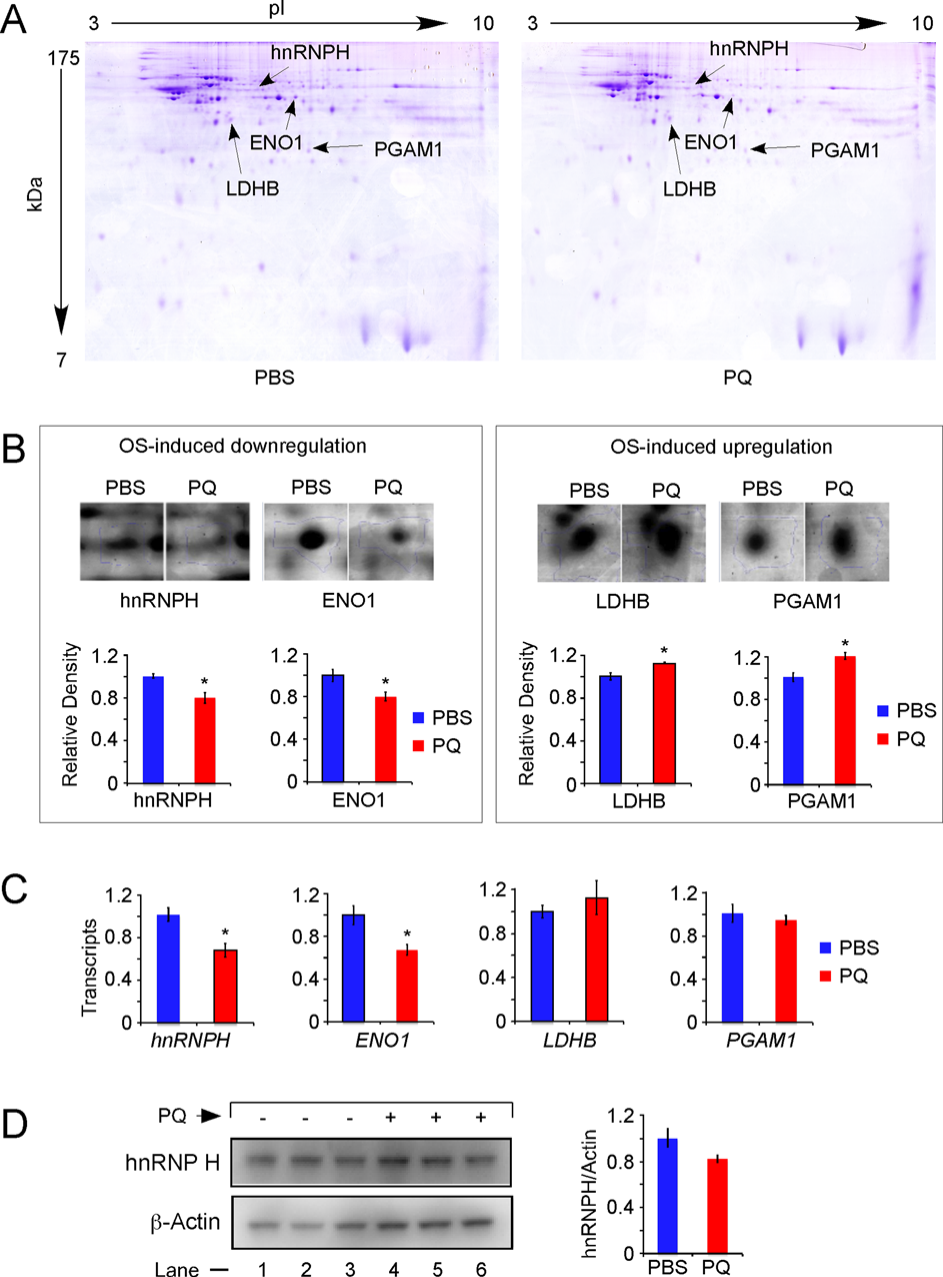
Proteome analysis of brain from PBS and PQ treated TG mice. PQ treatment and tissue collections were the same as described in Fig 2A. (A) Two-dimensional iso-electric focusing and SDS polyacrylamide gel electrophoresis (2D-IEF-SDS-PAGE) of PBS and PQ-treated TG brain. Differentially expressed proteins are indicated by arrows. (B) Amplified spots from panel A were identified by MALDI-TOF MS analysis. Target proteins are magnified. The bar diagrams show the relative expression of identified proteins in the absence and presence of PQ. Error bars represent standard error. Stars above bars indicate statistical significance (*, *P <* 0.05). (C) Levels of various transcripts determined by QPCR in samples used in panel A. (D) Western blot showing the effect of PQ treatment on the level of hnRNP H in the brain of TG mice. PQ treatment and tissue collection were the same as described in Fig 2A. Details of Western blot are the same as described in Fig 6.

To capture novel regulatory factors that are likely to affect level of SMN under OS conditions, we employed a two-dimensional (2D) gel electrophoreses of lysates prepared from TG brains harvested at 24 h post PQ treatment. We performed two independent experiments to ensure the reproducibility of our findings. After comparing the results of 2D gel electrophoresis, we investigated four protein spots that were significantly altered in brains subjected to OS (Fig 7A). We analyzed these proteins by mass spectrometry using MALDI-TOF and LC/MS (S2 Table). These proteins were identified as lactate dehydrogenase B (LDHB), phosphoglycerate mutase 1 (PGAM1), alpha-enolase 1 (ENO1) and hnRNP H. While LDHB and PGAM1 were found to be upregulated, ENO1 and hnRNP H were found to be downregulated (Fig 7B). To determine whether the altered expression of the above proteins was due to changes in transcription, we performed QPCR. Consistent with the OS-induced downregulation of ENO1 and hnRNP H proteins, we observed a small but statistically significant decrease in their transcripts (Fig 7C). However, we did not capture a significant change in *Ldhb* and *Pgam1* transcripts in PQ-treated brain, suggesting that posttranslational modification(s) cause differential expression of LDHB and PGAM1 (Fig 7C). Considering that ENO1, LDHB and PGAM1 are involved in glycolysis and/or gluconeogesis pathways, their alteration during OS was not surprising. In particular, downregulation of ENO1 in striatum of PQ-treated mice mimicking Parkinson’s disease has been previously reported [66].

A recent study precluded the role of hnRNP H in *SMN* exon 7 splicing, since the depletion of this protein did not affect splicing of exon 7 [67]. However, the effect of hnRNP H has not been tested in splicing of other *SMN* exons. Moreover, the conditions of OS could cause post-translational modifications in hnRNP H. Such modified hnRNP H can then affect splicing of *SMN2* exons. Having detected the OS-induced downregulation of hnRNP H in our proteome analysis, we performed Western blot to determine whether the conditions of OS cause a general decline in expression of hnRNP H. We did not capture a statistically significant decrease in the level of hnRNP H in brain of TG mice treated with PQ (Fig 7D). These results suggest that the change in the level of hnRNP H captured in proteome analysis is likely due to post-translational modification(s). To independently validate these findings in the context of a human cell line, we performed experiments in SH-SY5Y cells as previously described [41]. Similar to the results obtained in TG brain, we observed no significant decrease in the level of hnRNP H in SH-SY5Y cells treated with PQ (S7 Fig). We also performed similar experiment in SMA patient fibroblasts (GM03813 cells) and observed no significant change in the level of hnRNP H in PQ-treated fibroblasts (S7 Fig).

## Discussion

Here we report a comprehensive analysis of OS-induced aberrant splicing of multiple exons of *SMN2*, an ubiquitously expressed human gene associated with SMA, which is the most frequent genetic cause of infant mortality. We employed a healthy transgenic (TG) mouse model to assess the tissue-specific effect of PQ-induced OS on splicing of various *SMN2* exons. We chose PQ based on its classical usage across different animal species to study the molecular mechanisms of OS [68–70]. In a most revealing manner, our results of MESDA, RT-PCR and QPCR demonstrated body-wide susceptibility of various *SMN2* exons to OS conditions in TG mice (Figs 1–4; S2 Fig). Barring testis, all tissues exposed to PQ showed near total disappearance of the FL transcript, while transcripts in which exon 7 was skipped alone or together with exon 5 were the major splice isoforms. Of note, while levels of ΔC5,7 transcript significantly increased in a tissue-independent manner, level of ΔC7 transcript was not significantly affected by OS (Fig 3). These results suggest that the OS-induced skipping of exon 7 triggered skipping of exon 5 or vice versa. The effect of OS on *SMN2* exon 3 splicing was also tissue-independent (Fig 3). The skipping of this exon predominantly occurred in combination with the skipping of other exon(s). Splicing of *Sbp2* exons 3/3a suggested that the conditions of OS could favor usage of an alternative splice site within an existing exon. However, none of the OS-induced *SMN2* splicing events was associated with an alternative splice site usage.

The results of MESDA indicate that expression of ΔC7 and ΔC5,7 transcripts in the liver of TG mice peaked at 8 h and 24 h post PQ treatment, respectively (Fig 1B). It is likely that early skipping of *SMN2* exon 7 in OS is brought about by sudden change in the posttranslational modifications and/or nuclear import/export of splicing factors, whereas delayed skipping of exon 5 is affected by subsequent changes, such as a slow rate of transcription elongation. Generally, a faster rate of transcription elongation favors exon skipping due to the lack of time for the recruitment of factors that define splice sites [71]. Our finding of an OS-induced reduction in the retention of intron 1, the largest *SMN* intron, might support that a slow elongating RNA polymerase II helps recruit critical splicing factor(s) for a fast removal of intron 1. However, a slow elongating RNA polymerase II may also recruit negative factors, leading to enhanced skipping of downstream exons. Consistently, a recent report supports recruitment of an inhibitory factor during slow transcription elongation leading to the skipping of exon 9 of CFTR [72]. It is possible that slow removal of intron 1 under normal conditions is designed to hold the partially spliced *SMN* transcripts in the nucleus until other introns are excised. However, it remains to be seen how the rate of *SMN* transcription affects the splicing of various *SMN* exons.

Transcription and splicing of a large number of genes are differently regulated in mammalian testis [73,74]. We have recently shown that the levels of Smn protein in testis of mice are more than a magnitude higher than in most tissues, including brain, spinal cord, heart, liver, lung and kidney [32]. It appears that the high level of SMN in human testis is maintained at least in part due to the presence of *SMN2* that goes through an adult-specific splicing switch to generate mostly FL transcripts [32]. While the mechanism of this unique splicing switch remains to be uncovered, we hypothesize that the high level of SMN in testis is necessary to maintain the sufficient levels of functional snRNPs for the heightened splicing activity during spermatogenesis. In addition, low SMN causes c-Jun NH2-terminal kinase (JNK) signaling pathway activation that is associated with the testicular toxicity [23,75,76]. Therefore, maintaining a high level of SMN in testis is critical for sustaining general testicular health. It is known that low SMN exacerbates skipping of *SMN2* exon 7 [77]. Hence, high SMN level might at least partly contribute to the prevention of OS-induced skipping of *SMN2* exons in testis. In addition, it is possible that splicing factors responsible for the splicing switch of *SMN2* in this organ are not affected by PQ-induced OS. It is also likely that the blood testis barrier is less permeable to PQ. Hence, PQ might not penetrate the testis in amount sufficient enough to produce a significant effect on splicing of the *SMN2* exons. Supporting this argument, an early study on PQ poisoning in Crete detected markedly less PQ accumulation in testis compared to other tissues, including kidney, liver and lung [78].

Promoters of human *SMN* and mouse *Smn* genes share several but not all motifs [79]. However, since none of the *Smn* exons showed skipping under the normal and OS conditions (Fig 2B), we hypothesize that human-specific promoter elements and/or cis-elements within transcribed pre-mRNA are the driving force of splicing regulation of *SMN* exons. While a direct role of a promoter element(s) on splicing of *SMN* exons is yet to be established, a number of exonic and intronic cis-elements that weaken the 3′ and 5′ ss of *SMN2* exon 7 have been implicated in skipping *SMN2* exon 7 [63]. It has also been shown that the sequestration of a negative cis-element by an 8-mer ASO prevents skipping of *SMN2* exon 7 under normal and OS conditions [41,80–82]. Hence, a correlation could be drawn between a suboptimal splice site and susceptibility to aberrant splicing under conditions of OS. Genome-wide analyses show the prevalence of R (purines) and G residues at the first and the last positions of human exons, respectively [53]. In our study, most exons susceptible to skipping induced by OS lacked the favorable residues at the first and the last positions of exons (Fig 5A). Lack of a G residue at the last position of an exon weakens the base pairing between U1 RNA and the 5′ ss. In several cases we examined, the RNA:RNA duplex formed between U1 RNA and the 5′ ss appeared to be weak due to less than six continuous base pairs (Fig 5A). Exons that were susceptible to inclusion induced by OS appeared to have favorable residues at the terminal exonic positions. Yet, the 5′ ss of most of these exons had poor base pairing with U1 RNA (Fig 5A). We have previously shown an inhibitory structural context as one of the limiting factors for skipping of *SMN2* exon 7 [83]. Most of the exons we examined had predicted stem-loop structures that sequester their 5′ ss (Fig 5C). We also observed enrichment of several motifs within OS-sensitive exons and their flanking intronic sequences (S5 Fig). The compositions of enriched motifs in skipping-prone exons were distinct from those in inclusion-prone exons. Future experiments will determine how these motifs cooperate to regulate alternative splicing under OS.

OS caused a small but statistically significant decrease in hSMN protein derived from *SMN2*. Due to the insertion of a protein degradation signal upon skipping of exon 7 [19], we were unable to detect proteins translated from aberrantly spliced *SMN2* transcripts, the majority of which lacked exon 7. Despite being translation compatible, none of the alternatively spliced variants of human *SMN* have been assigned any specific function. It is possible that *SMN2* transcripts generated under OS play some role. For example, the short *SMN2* isoforms could act as long-noncoding RNAs (lncRNAs). One of the critical functions of lncRNAs is to serve as competing RNAs to sequester microRNAs and RNA-binding proteins [84,85]. Considering FL and truncated *SMN2* transcripts generated under OS possess identical 3′ untranslated regions (3′UTRs), they might compete for the same factors that regulate translation via 3′UTRs. Therefore, the reason for only a small reduction in FL SMN level despite a significant drop in level of FL transcript could be the enhanced translation of FL transcripts due to the 3′UTRs of the short *SMN2* transcripts generated under OS acting as a “sponge” for the inhibitory microRNA.

Even though we did not capture a major shift in OS-induced expression of several splicing factors, we did observe a small but significant decrease in levels of TIA1 and hnRNP A2. These proteins have the opposite effects on *SMN2* exon 7 splicing [82]. While TIA1 promote inclusion of exon 7, hnRNP A2 promotes exclusion of exon 7. Although not statistically significant, we also observed a noticeable increase in the level of SRp55. The role of SRp55 has been implicated in the inhibition of the 5′ ss of exon 3 of HIV-1 vpr mRNA [65]. Recently, SRp55 was shown to inhibit *SMN* exon 7 splicing [67]. Changes in the levels of splicing factors described in this study do not fully capture the overall mechanistic aspects by which splicing of various *SMN* exons are regulated under normal and OS conditions. Future studies should focus on how splicing of specific *SMN* exons are regulated. Despite the fact that an overwhelming OS-induced skipping of *SMN2* exon 7 (together with exon 5) was recorded in most tissues, it wouldn’t be surprising that an entirely different set of splicing factors are involved in splicing of these exons in different tissues. Evidence supporting this argument comes from our parallel experiment with *Sbp2*. Every tissue examined showed a different pattern of *Sbp2* exon 3/3a usage under OS conditions (Fig 2C).

Our proteome analysis revealed downregulation of hnRNP H as one of the consequences of PQ-induced OS (Fig 7). Our follow-up experiments suggested that the differential expression of hnRNP H captured in the proteome analysis is likely due to a posttranslational modification(s). In addition to its effect on splicing, there are other mechanisms by which hnRNP H could regulate level of SMN under normal and OS conditions. For instance, the involvement of hnRNP H in regulation of 3′-end processing has been shown for human β-globin and p53 transcripts [86,87]. Interestingly, a transcriptome-wide analysis employing CLIP-seq revealed direct interaction of hnRNP H with *SMN* exon 8 [88]. Future studies will determine if hnRNP H plays any role in splicing and/or 3′-end processing of *SMN* transcripts. While this is the first in vivo study to show the effect of a single high dose of an OS-inducing agent on splicing of various exons of *SMN* in different tissues, outcomes are likely to vary if animals are exposed to an OS-inducing agent for a longer duration. Also, the background and/or the age group of mice are likely to influence the effect of OS on splicing of various *SMN* exons. Addressing these questions will provide a better understanding of the role of SMN in diseases caused by OS.

## Conclusion

In conclusion, our findings demonstrate the susceptibility of various *SMN2* exons to skipping under the conditions of OS and implicate multiple cis-elements and transacting factors in splicing regulation of different *SMN* exons under normal and OS conditions.

## Supporting Information

S1 Fig Effect of PQ treatment on animal survival as well as on the level of molecular markers for OS. (A) Effect of PQ on survival of TG mice. Kaplan-Meier survival analysis of TG mice injected with PQ (70 mg/kg). N indicates number of mice used for this study. (B) Western blot analysis demonstrating the levels of 4-Hydroxynonenal adducts of histidine residues in response to PQ treatment. Brain samples were collected at 0 h (control) and 8 h post PQ injection. We used 35 μg of total protein for each sample. β-actin was used as a loading control. Densitometric analysis of Western blots is shown in the right panel. Stars indicate statistical significance (*, *P*< 0.05). AU, arbitrary units. (C) Western blot analysis demonstrating the expression of total and phosphorylated AKT (pAKT) protein in brain of control or PQ-treated animals. Brain samples were collected at 0 h (control) and 8 h post PQ injection. We used 50 μg of total protein for each sample. Names of probed proteins are indicated on the left of each blot. Sizes of proteins based on molecular weight markers are indicated on the right of each blot. β-actin was used as a loading control. Densitometric analysis of Western blots is shown in the right panel. Bars represent the relative expression of pAKT versus AKT proteins. Stars indicate statistical significance (*, *P*< 0.05). AU, arbitrary units.
(TIF)

S2 Fig Effect of PQ treatment on splicing of various exons of human SMN2 and mouse Smn in different tissues of TG mice. (A) Diagrammatic representation of exon-intron organization of *SMN2* and *Smn*. Exons are indicated as colored boxes, while introns are shown as lines. Sizes of introns and exons are given as well. (B) In vivo splicing pattern of exon 7 in *SMN2* and *Smn* in different tissues of mice injected with PBS (-) or PQ (+). Names of tissues are indicated at the top of the gel. The upper band corresponds to a product that includes exon 7, while the lower band corresponds to a product in which exon 7 is skipped. The percentage of exon 7 skipping is given at the bottom of the gel. It was calculated from the total value of exon 7-included and exon 7-skipped products. *SMN2* products were amplified using human-specific primers annealing to exon 6 (N-24) and exon 8 (P2-2). For *Smn* mouse-specific primers annealing to exons 6 (5SmnE6) and 8 (3SmnE8) were used. Abbreviations: E7, exon 7. (C) In vivo splicing pattern of exon 5 in *SMN2* and *Smn* in different tissues of mice injected with PBS (-) or PQ (+). Descriptions are the same as in panel A. *SMN2* products were amplified using human-specific primers annealing to exon 4 (5SMNE4) and exon 6 (3SMNE6). For *Smn*, mouse-specific primers annealing to exons 4 (5SmnE4) and 6 (3SmnE6) were used. Abbreviations: E5, exon 5. (D) In vivo splicing pattern of exon 3 of *SMN2* and *Smn* in different tissues of mice injected with PBS (-) or PQ (+). Descriptions are the same as in panel A. Human-specific primers annealing to exons 1 (5SMNE1) and 4 (3SMNE4) and mouse-specific primers annealing to exons 1 (5SmnE1) and 4 (3SmnE4) were used for amplification of *SMN2* and *Smn* products, respectively. Abbreviations: E3, exon 3.
(TIF)

S3 Fig Alignment of sequences corresponding to mouse Sbp2 (mSbp2) and human SBP2 (hSBP2) exon 3 and its flanking intronic sequences. (A) Sequence alignment was done using MacVector software. Nucleotides numbered as -1, 1 and +1 correspond to the last position of intron 2, the beginning of exon 3 and the start of intron 3, respectively. Exon 3 is split into exon 3a (purple) and exon 3b (green). Arrows indicate the splice sites. Stars indicate positions of sequence identity. (B) Recognition of the 5′ ss of *mSbp2* exon 3 by U1 snRNA. The 5′ ss:U1 snRNA base pairing is indicated by the vertical lines. (C) Secondary structure predicted to form at the 5′ ss of *mSbp2* exon 3. Uppercase letters correspond to nucleotides of exon 3, lowercase letter to nucleotides of intron 3. (D) The effect of PQ on splicing of *SBP2*. Diagrammatic representation of *SBP2* alternative splicing under normal and oxidative-stress conditions is shown in the left panel. In vivo splicing pattern of *SBP2* in different cell lines in the presence (+) or absence (-) of PQ is shown in the right panel. Names of cell lines used are given at the top of the gel. The identity of splice isoforms is indicated on the right of the gel. To amplify *SBP2* splice isoforms human-specific primers annealing to exon 1 (5hSBP2E1) and 4 (3hSBP2E4) were employed.
(TIF)

S4 Fig Effect of PQ on intron retention during SMN2 splicing. (A) Diagrammatic representation of intron-retained *SMN2* transcripts. Exons are indicated as colored boxes. Alternatively spliced exons 3, 5 and 7 are indicated as colored ovals. Introns are shown as lines. Name of the splice isoforms are given on the left of each diagram. Of note, only one out of many splice variants with a specific retained intron is shown. Annealing positions and names of primers used for QPCR are indicated. To detect intron retention, we used a primer pair in which a forward primer annealed within the exon and a reverse primer annealed to the intron of interest in the vicinity of the 5′ ss. cDNA was reverse transcribed using random primers and total RNA prepared from brain of PBS and PQ treated animals. (B) Quantification of intron retention by QPCR. Expression levels relative to PBS treated brain for each isoform were normalized to value of 1. Error bars represent standard error. Stars above PQ bars indicate statistical significance (*, *P < 0*.*05*).
(TIF)

S5 Fig Analyses of motifs enriched within exons whose splicing was sensitive to OS. Intronic sequences that flank these exons were included in the analysis as well. (A) Enriched motifs of OS-sensitive exons that showed enhanced skipping under PQ-induced OS. (B) Enriched motifs of OS-sensitive exons that showed enhanced inclusion under PQ-induced OS.
(TIF)

S6 Fig Efficiency of translation of various SMN2 isoforms generated by alternative splicing. (A) Diagrammatic representation of SMN domain structure. Exons, the number of amino acids they encode, protein domains and their functions are all indicated. The UTRs, the start and the stop codons are marked. Abbreviations: UTR, untranslated region. (B) Diagrammatic representation of FLAG-tagged *SMN* splice isoforms cloned into pCI-NEO mammalian expression vector (S3 Table). Names of the protein isoforms as well as their expected sizes are shown on the right of each construct. Exons are shown as colored boxes. Broken lines indicate skipped exons. (C) Expression of FLAG-tagged SMN protein isoforms in HeLa cells transfected with mammalian expression vectors described in panel B as determined by Western blot. Name of each protein isoform is indicated at the top of the blot. Names of probed peptide/proteins are indicated on the left. β-actin was used as a loading control.
(TIF)

S7 Fig Effect of PQ treatment on levels of hnRNP H protein in human cell lines. Pre-plated SH-SY5Y (A) and GM03813 (SMA patient fibroblasts) (B) cells were treated with 1 mM of PQ for 24 hours, after which the cells were collected for preparation of protein lysates. Top panels, labeled MESDA, indicate multiple-exon-skipping events in endogenous *SMN1/SMN2* genes in the absence (-) or presence (+) of PQ. Spliced isoforms were amplified using primers annealing to exon 1 (5SMNE1TSS) and exon 8 (3E8-25). The identities of spliced isoforms are marked on the left of the gel. Bottom panel, labeled Western blot, shows the levels of hnRNP H protein in SH-SY5Y (A) and GM03813 (B) cells in the absence (-) or presence (+) of PQ. 20 and 10 μg of proteins were loaded from SH-SY5Y and GM03813 lysates, respectively. β-actin was used as a loading control. Densitometric quantifications are given as well. Abbreviations: FL, full length.
(TIF)

S1 Table List of primers used for PCR and QPCR. (DOCX)

S2 Table A list of altered proteins from PQ treated TG brain. (DOCX)

S3 Table Description and GenBank accession numbers of mammalian expression vectors. (DOCX)
