# Supplementary figures and images for: Oxidative Stress Triggers Body-Wide Skipping of Multiple Exons of the Spinal Muscular Atrophy Gene
Source: PLoS One. 2016 Apr 25;11(4):e0154390. doi: 10.1371/journal.pone.0154390 (PMC4844106; doi:10.1371/journal.pone.0154390)

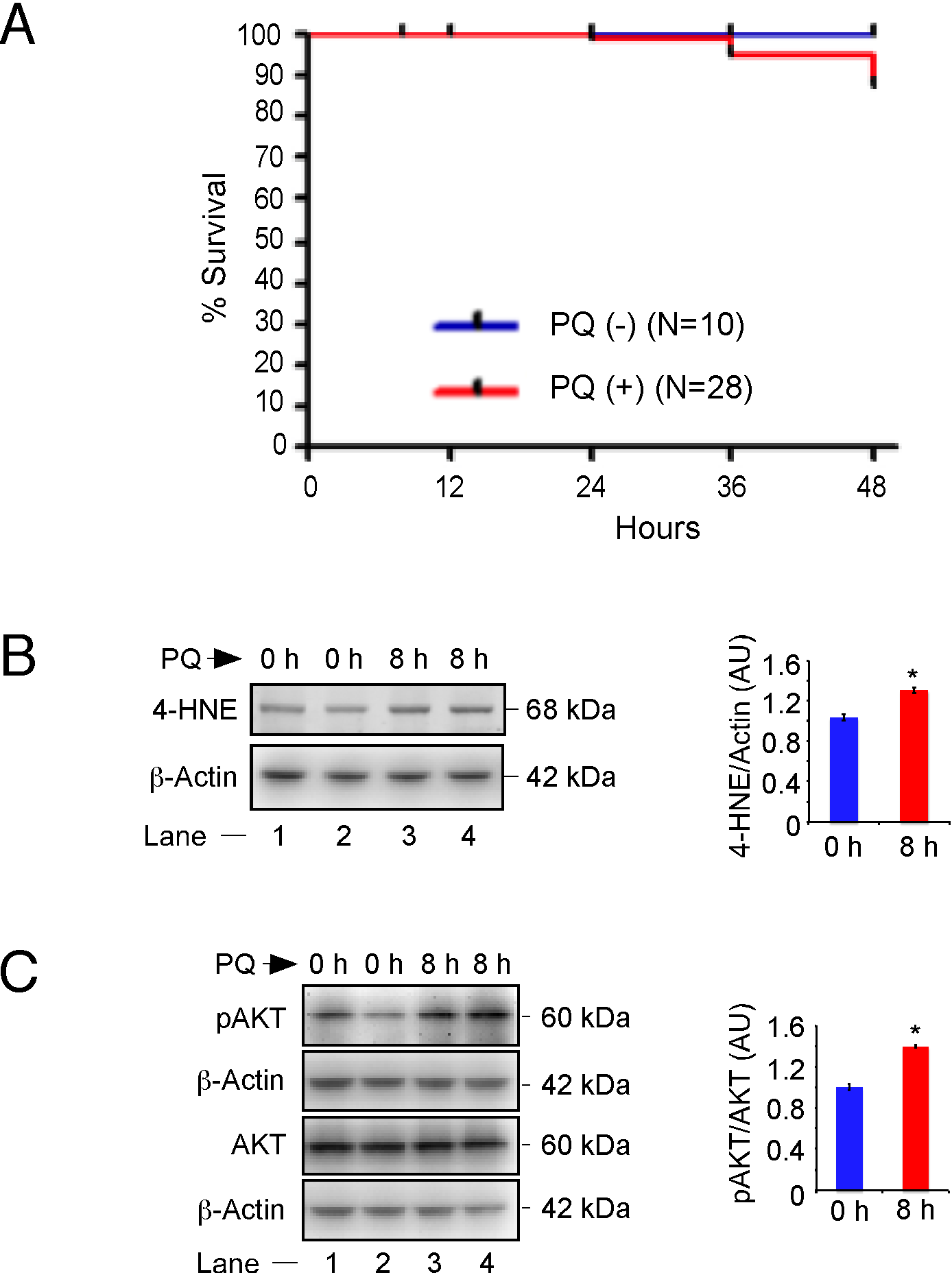

Supplement: S1 Fig — (A) Effect of PQ on survival of TG mice. Kaplan-Meier survival analysis of TG mice injected with PQ (70 mg/kg). N indicates number of mice used for this study. (B) Western blot analysis demonstrating the levels of 4-Hydroxynonenal adducts of histidine residues in response to PQ treatment. Brain samples were collected at 0 h (control) and 8 h post PQ injection. We used 35 μg of total protein for each sample. β-actin was used as a loading control. Densitometric analysis of Western blots is shown in the right panel. Stars indicate statistical significance (*, P< 0.05). AU, arbitrary units. (C) Western blot analysis demonstrating the expression of total and phosphorylated AKT (pAKT) protein in brain of control or PQ-treated animals. Brain samples were collected at 0 h (control) and 8 h post PQ injection. We used 50 μg of total protein for each sample. Names of probed proteins are indicated on the left of each blot. Sizes of proteins based on molecular weight markers are indicated on the right of each blot. β-actin was used as a loading control. Densitometric analysis of Western blots is shown in the right panel. Bars represent the relative expression of pAKT versus AKT proteins. Stars indicate statistical significance (*, P< 0.05). AU, arbitrary units. (TIF) [file pone.0154390.s001.tif]

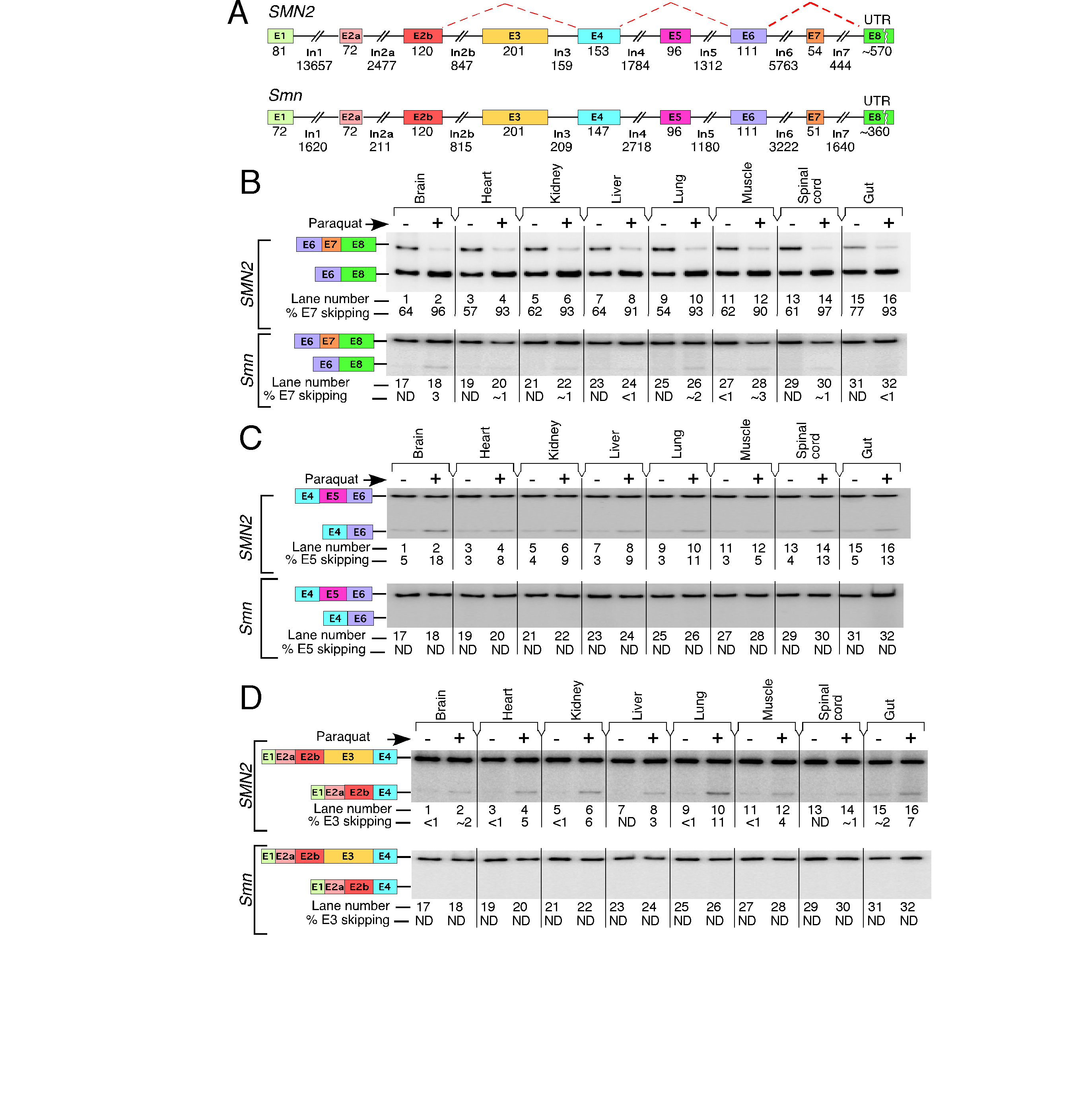

Supplement: S2 Fig — (A) Diagrammatic representation of exon-intron organization of SMN2 and Smn. Exons are indicated as colored boxes, while introns are shown as lines. Sizes of introns and exons are given as well. (B) In vivo splicing pattern of exon 7 in SMN2 and Smn in different tissues of mice injected with PBS (-) or PQ (+). Names of tissues are indicated at the top of the gel. The upper band corresponds to a product that includes exon 7, while the lower band corresponds to a product in which exon 7 is skipped. The percentage of exon 7 skipping is given at the bottom of the gel. It was calculated from the total value of exon 7-included and exon 7-skipped products. SMN2 products were amplified using human-specific primers annealing to exon 6 (N-24) and exon 8 (P2-2). For Smn mouse-specific primers annealing to exons 6 (5SmnE6) and 8 (3SmnE8) were used. Abbreviations: E7, exon 7. (C) In vivo splicing pattern of exon 5 in SMN2 and Smn in different tissues of mice injected with PBS (-) or PQ (+). Descriptions are the same as in panel A. SMN2 products were amplified using human-specific primers annealing to exon 4 (5SMNE4) and exon 6 (3SMNE6). For Smn, mouse-specific primers annealing to exons 4 (5SmnE4) and 6 (3SmnE6) were used. Abbreviations: E5, exon 5. (D) In vivo splicing pattern of exon 3 of SMN2 and Smn in different tissues of mice injected with PBS (-) or PQ (+). Descriptions are the same as in panel A. Human-specific primers annealing to exons 1 (5SMNE1) and 4 (3SMNE4) and mouse-specific primers annealing to exons 1 (5SmnE1) and 4 (3SmnE4) were used for amplification of SMN2 and Smn products, respectively. Abbreviations: E3, exon 3. (TIF) [file pone.0154390.s002.tif]

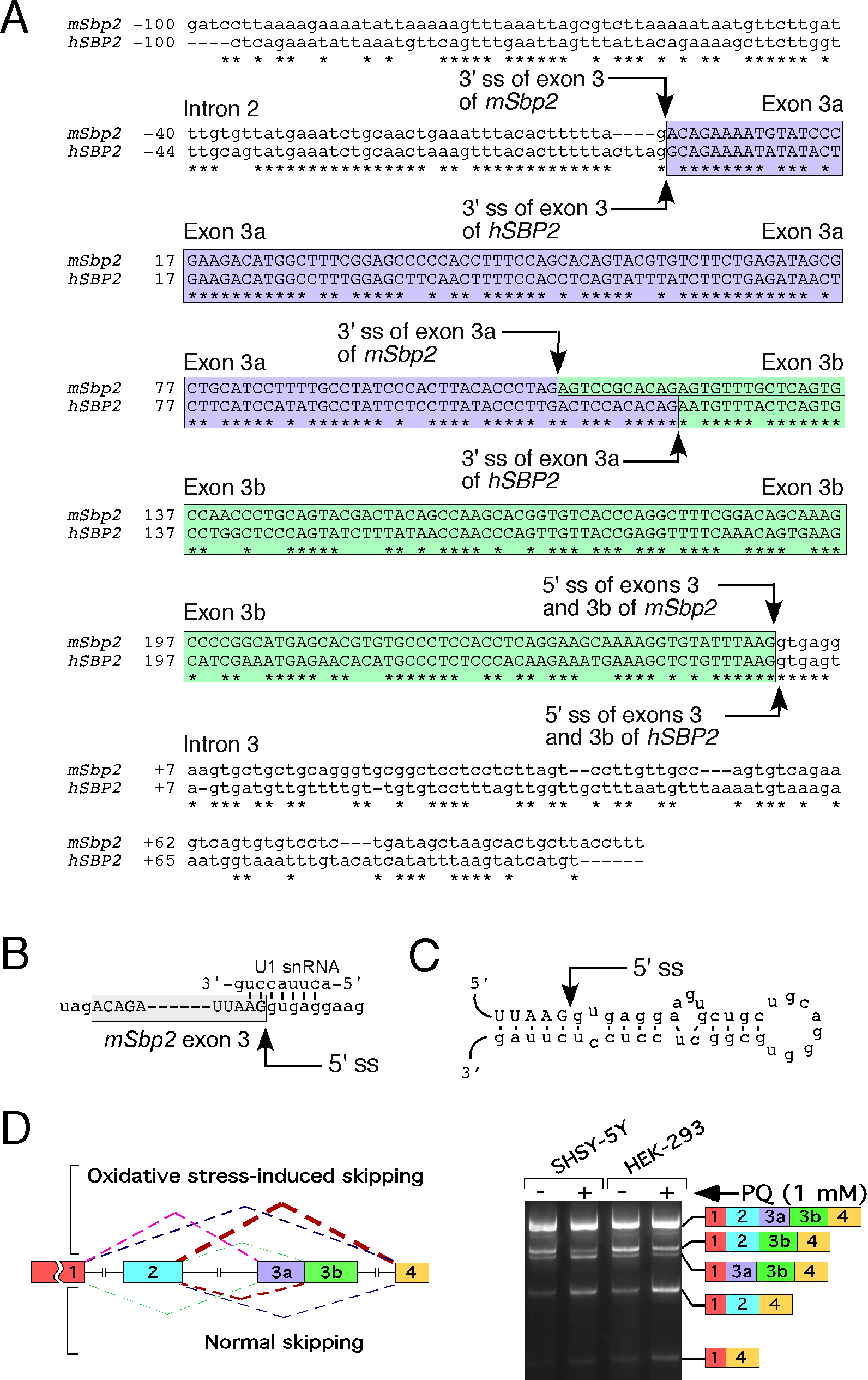

Supplement: S3 Fig — (A) Sequence alignment was done using MacVector software. Nucleotides numbered as -1, 1 and +1 correspond to the last position of intron 2, the beginning of exon 3 and the start of intron 3, respectively. Exon 3 is split into exon 3a (purple) and exon 3b (green). Arrows indicate the splice sites. Stars indicate positions of sequence identity. (B) Recognition of the 5′ ss of mSbp2 exon 3 by U1 snRNA. The 5′ ss:U1 snRNA base pairing is indicated by the vertical lines. (C) Secondary structure predicted to form at the 5′ ss of mSbp2 exon 3. Uppercase letters correspond to nucleotides of exon 3, lowercase letter to nucleotides of intron 3. (D) The effect of PQ on splicing of SBP2. Diagrammatic representation of SBP2 alternative splicing under normal and oxidative-stress conditions is shown in the left panel. In vivo splicing pattern of SBP2 in different cell lines in the presence (+) or absence (-) of PQ is shown in the right panel. Names of cell lines used are given at the top of the gel. The identity of splice isoforms is indicated on the right of the gel. To amplify SBP2 splice isoforms human-specific primers annealing to exon 1 (5hSBP2E1) and 4 (3hSBP2E4) were employed. (TIF) [file pone.0154390.s003.tif]

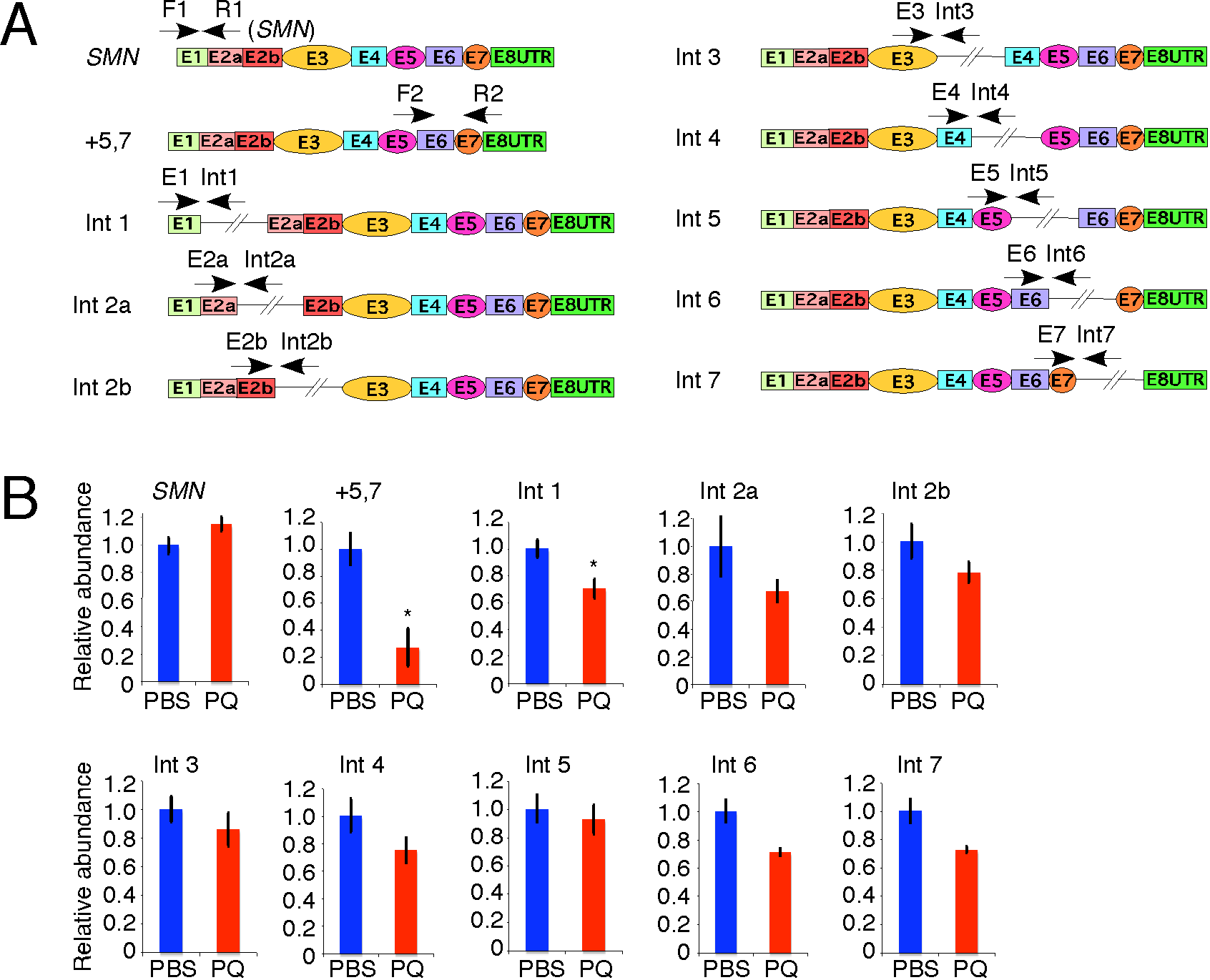

Supplement: S4 Fig — (A) Diagrammatic representation of intron-retained SMN2 transcripts. Exons are indicated as colored boxes. Alternatively spliced exons 3, 5 and 7 are indicated as colored ovals. Introns are shown as lines. Name of the splice isoforms are given on the left of each diagram. Of note, only one out of many splice variants with a specific retained intron is shown. Annealing positions and names of primers used for QPCR are indicated. To detect intron retention, we used a primer pair in which a forward primer annealed within the exon and a reverse primer annealed to the intron of interest in the vicinity of the 5′ ss. cDNA was reverse transcribed using random primers and total RNA prepared from brain of PBS and PQ treated animals. (B) Quantification of intron retention by QPCR. Expression levels relative to PBS treated brain for each isoform were normalized to value of 1. Error bars represent standard error. Stars above PQ bars indicate statistical significance (*, P < 0.05). (TIF) [file pone.0154390.s004.tif]

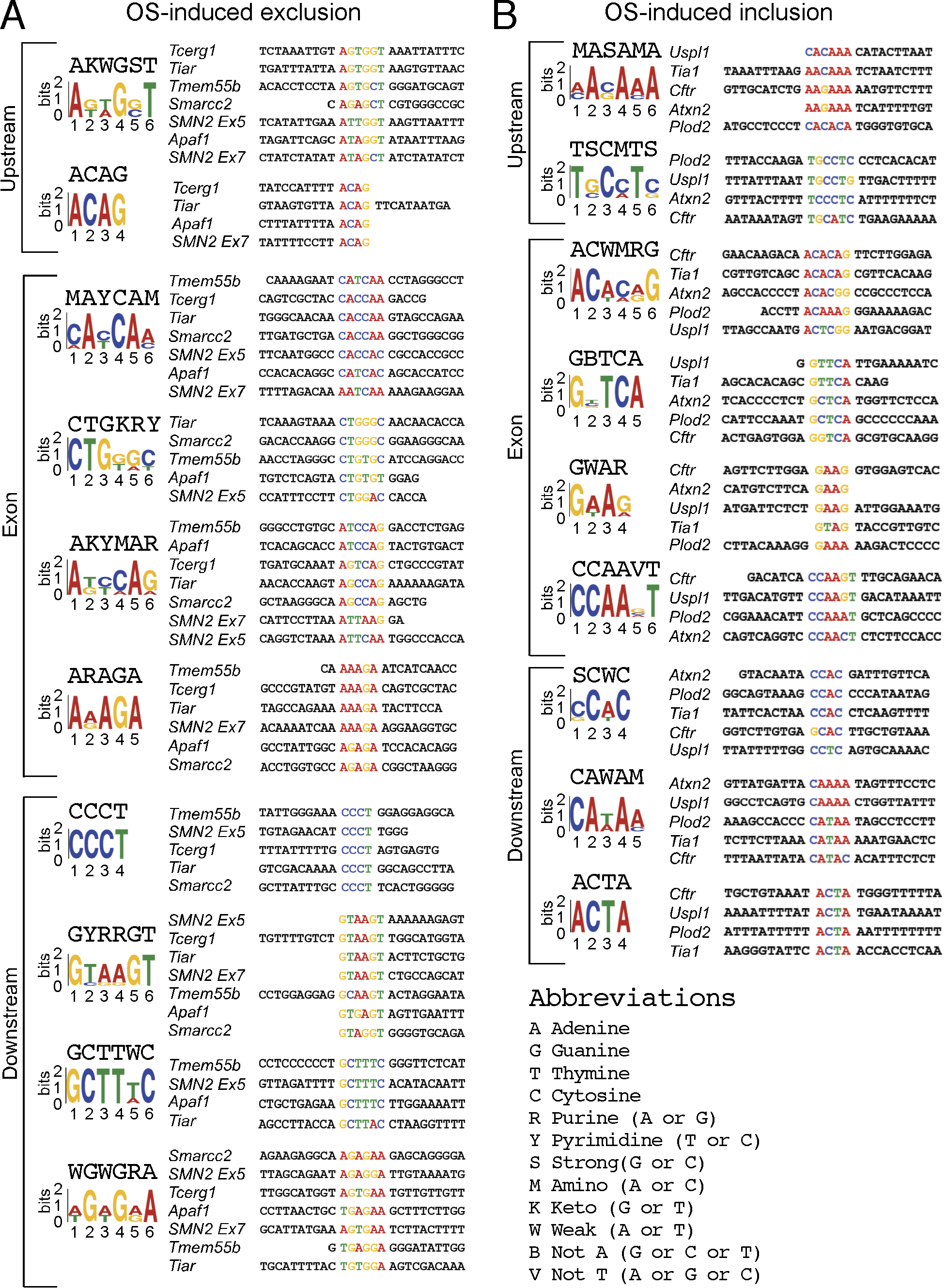

Supplement: S5 Fig — Intronic sequences that flank these exons were included in the analysis as well. (A) Enriched motifs of OS-sensitive exons that showed enhanced skipping under PQ-induced OS. (B) Enriched motifs of OS-sensitive exons that showed enhanced inclusion under PQ-induced OS. (TIF) [file pone.0154390.s005.tif]

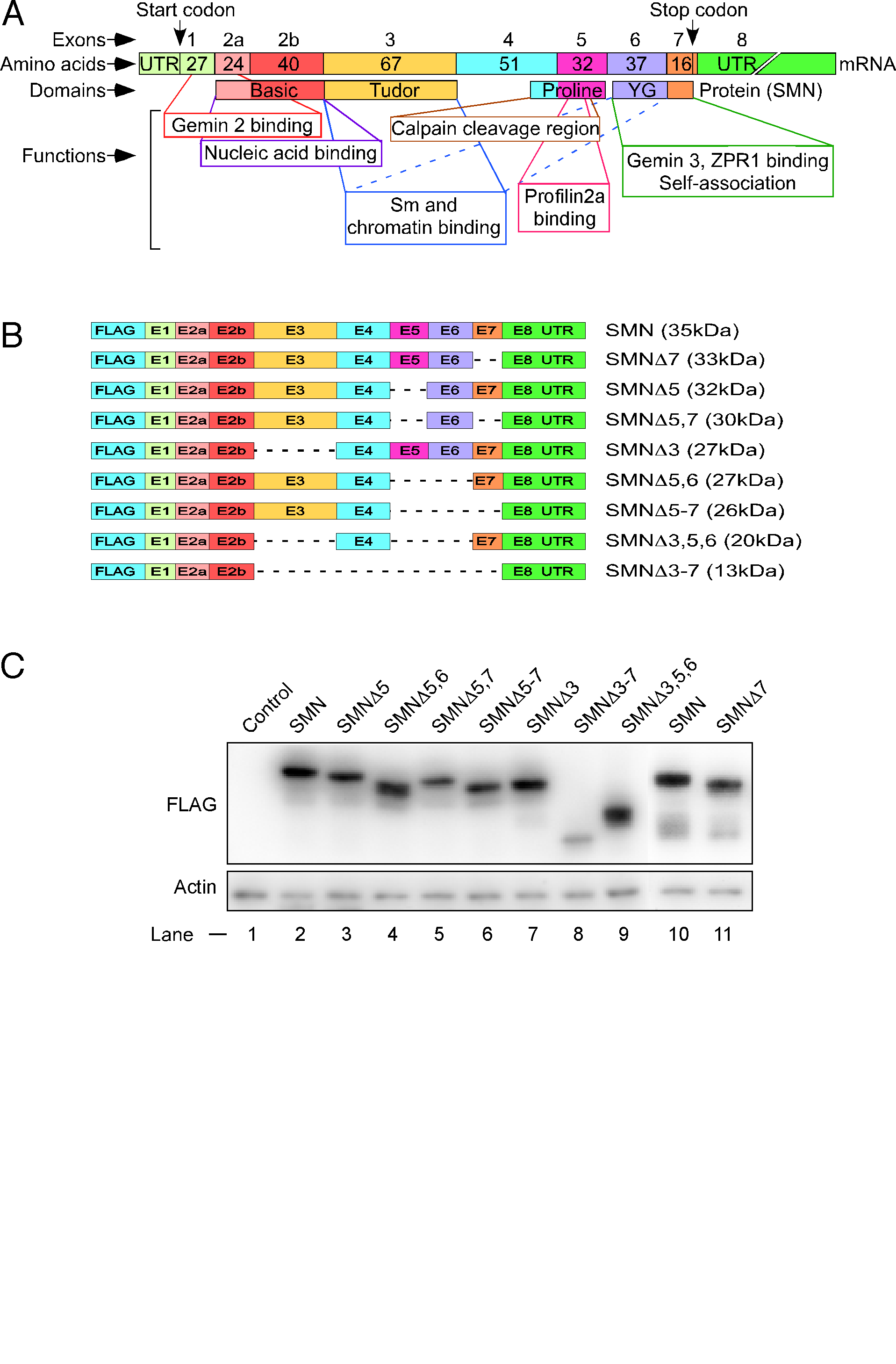

Supplement: S6 Fig — (A) Diagrammatic representation of SMN domain structure. Exons, the number of amino acids they encode, protein domains and their functions are all indicated. The UTRs, the start and the stop codons are marked. Abbreviations: UTR, untranslated region. (B) Diagrammatic representation of FLAG-tagged SMN splice isoforms cloned into pCI-NEO mammalian expression vector (S3 Table). Names of the protein isoforms as well as their expected sizes are shown on the right of each construct. Exons are shown as colored boxes. Broken lines indicate skipped exons. (C) Expression of FLAG-tagged SMN protein isoforms in HeLa cells transfected with mammalian expression vectors described in panel B as determined by Western blot. Name of each protein isoform is indicated at the top of the blot. Names of probed peptide/proteins are indicated on the left. β-actin was used as a loading control. (TIF) [file pone.0154390.s006.tif]

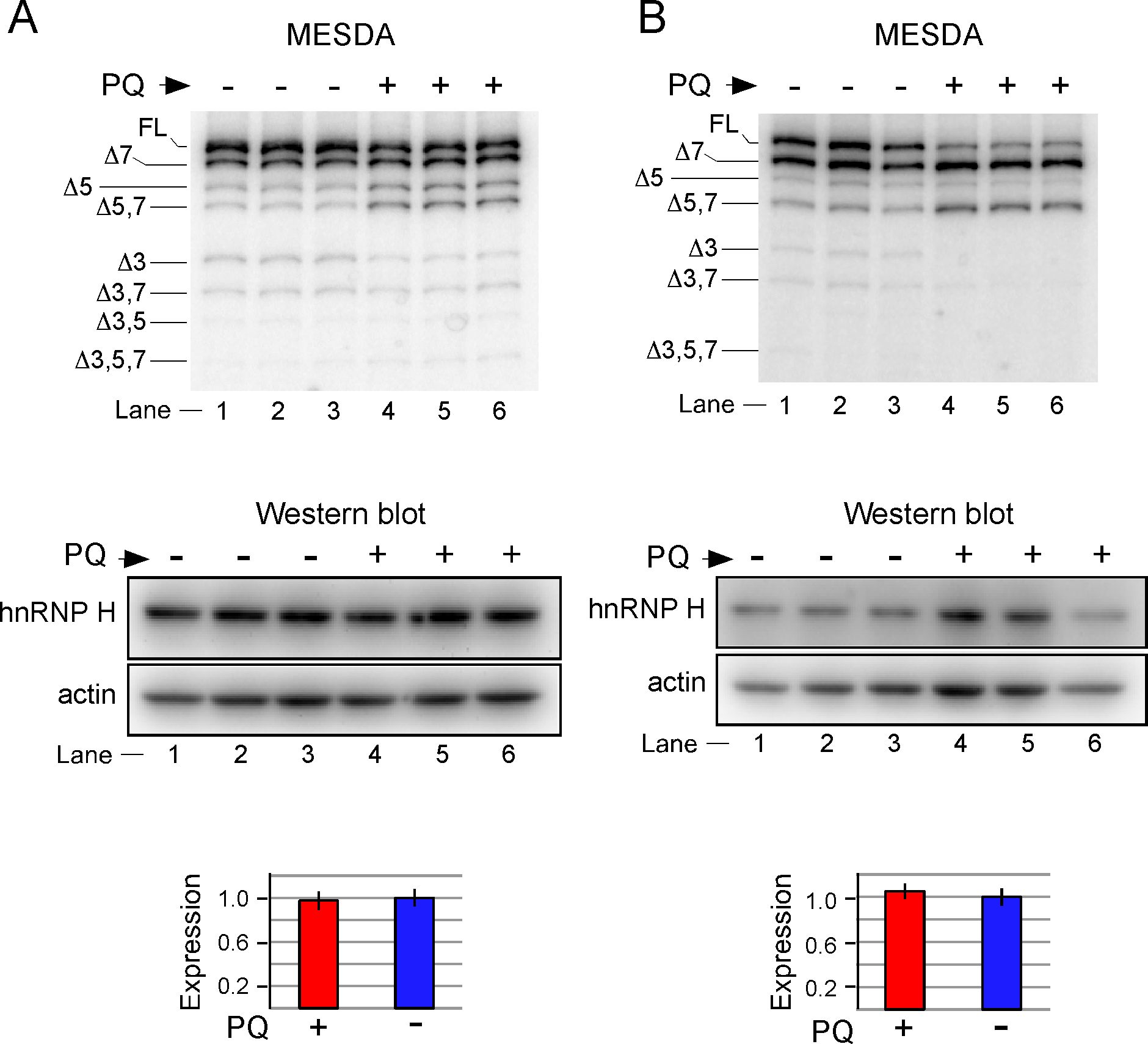

Supplement: S7 Fig — Pre-plated SH-SY5Y (A) and GM03813 (SMA patient fibroblasts) (B) cells were treated with 1 mM of PQ for 24 hours, after which the cells were collected for preparation of protein lysates. Top panels, labeled MESDA, indicate multiple-exon-skipping events in endogenous SMN1/SMN2 genes in the absence (-) or presence (+) of PQ. Spliced isoforms were amplified using primers annealing to exon 1 (5SMNE1TSS) and exon 8 (3E8-25). The identities of spliced isoforms are marked on the left of the gel. Bottom panel, labeled Western blot, shows the levels of hnRNP H protein in SH-SY5Y (A) and GM03813 (B) cells in the absence (-) or presence (+) of PQ. 20 and 10 μg of proteins were loaded from SH-SY5Y and GM03813 lysates, respectively. β-actin was used as a loading control. Densitometric quantifications are given as well. Abbreviations: FL, full length. (TIF) [file pone.0154390.s007.tif]
