# Supplementary material for: Oxidative Stress Triggers Body-Wide Skipping of Multiple Exons of the Spinal Muscular Atrophy Gene
Source: PLoS One. 2016 Apr 25;11(4):e0154390. doi: 10.1371/journal.pone.0154390 (PMC4844106; doi:10.1371/journal.pone.0154390)
Supplement: S1 Table — (DOCX) [file pone.0154390.s008.docx]

**S1 Table.** List of primers used for PCR and QPCR

| No. | Name | Type | Sequence (5′ to 3′) | Annealing site |
| --- | --- | --- | --- | --- |
| 1 | 5SMNE2B | Forward | GAATACTGCAGCTTCCTTACAACAG | E2B |
| 2 | P2-2 | Reverse | CTTCCTTTTTTCTTTCCCAACAC | E8 |
| 3 | 3E8-Dde | Reverse | CTACAACACCCTTCTCACAGCTC | E8 |
| 4 | 5SmnE1 | Forward | TGGGCAGTGGCGGAGCGGGC | E1 |
| 5 | 3SmnE8 | Reverse | ACCCCATCTCCTGAGACAGAGC | E8 |
| 6 | N-24 | Forward | CCAGATTCTCTTGATGATGCTGATGCTTTGGG | E6 |
| 7 | 5SmnE6 | Forward | GAGTGGCTACCACACTGGCTAC | E6 |
| 8 | 3SmnE8 | Reverse | CACGCTCTGCTGCTGACTTAGG | E8 |
| 9 | 5SMNE4 | Forward | GGCCAAGACTGGGACCAGG | E4 |
| 10 | 3SMNE6 | Reverse | CATATAATAGCCAGTATGATAGCC | E6 |
| 11 | 5SmnE4 | Forward | CGCTCAGAAGTAAAGCACACAGCAAG | E4 |
| 12 | 3SmnE6 | Reverse | GAGATTAGCATACTGCCCAGGG | E6 |
| 13 | 5SMNE1 | Forward | GAGCAGCGGCGGCAGTGGTG | E1 |
| 14 | 3SMNE4 | Reverse | TCACTTTCATCTGTTGAAACTTGG | E4 |
| 15 | 5SmnE1 | Forward | GTGGCGGAGCGGGCTCCGAG | E1 |
| 16 | 3SmnE4 | Reverse | CTTGCTGTGTGCTTTACTTCTGAGCG | E4 |
| 17 | 5FLAG | Forward | GATGACAAGACGCGTGCGAT |  |
| 18 | 5Ex1 | Forward | CGCGGGTTTGCTATGGCGAT | E1 |
| 19 | 3SMNE8 | Reverse | TAGTGCTGCTCTATGCCAGCATTTC | E8 |
| 20 | 5mTiar | Forward | CTCTCCAGAGATGTGACAGAAGTC | E2 |
| 21 | 3mTiar | Reverse | AATTTCTGGACTCAAATCCCC | E5 |
| 22 | 5mTmem55b | Forward | GCACCTCCAGGAAAGAAGTATGTC | E3 |
| 23 | 3mTmem55b | Reverse | CATCGTGCCAAGGTTCGGTC | E5 |
| 24 | 5mTcerg1 | Forward | CACCCACAACACAAGACCAGACC | E5 |
| 25 | 3mTcerg1 | Reverse | CCACTCAGAAACTGCTGTTGCCC | E7 |
| 26 | 5mSmarcc2 | Forward | CCTGGAACAGTGGGGTCTTATTAAC | E17 |
| 27 | 3mSmarcc2 | Reverse | CAGAGTCTCCTGCTCCGTCCATTC | E20 |
| 28 | 5SMNE1 | Forward | CCAAATCTGCTCCATGGAACTCTTTTC | E4 |
| 29 | 3SMNE4 | Reverse | GCATCAGCATCATCAAGAGAATCTGG | E6 |
| 30 | 5mApaf | Forward | GATGTGAGATCAGCAAACGAGAGG | E17 |
| 31 | 3mApaf | Reverse | CTAAAGGTGGCCGACTGCAGAG | E19 |
| 32 | 5mTia1 | Forward | GCCCAAGACTCTATACGTCGG | E1 |
| 33 | 3mTia1 | Reverse | GGTGCAAACGCTGCTTTGATG | E6 |
| 33 | 5mPlod2 | Forward | CCGATCTGAGATGAATGAAAGGAAC | E13 |
| 34 | 3mPlod2 | Reverse | TGCCAGAAGTCATTGTTAAGATGGG | E15 |
| 35 | 5mUspl1 | Forward | GGAGTTCGGGTCCACTG | E1 |
| 36 | 3mUspl1 | Reverse | CTTGCCTTTCGCTCTACAAG | E3 |
| 37 | 5mAtxn2 | Forward | TCAAGAGCTGCTTCTCACA | E9 |
| 38 | 3mAtxn2 | Reverse | AGGAGCAGCTGCTTCAC | E11 |
| 39 | 5mCftr | Forward | GTTCTTGGAGAAGGTGGAGTC | E11 |
| 40 | 3mCftr | Reverse | CAGAGGAATCGTCTACTGAGAACC | E13 |
| 41 | 5mSbp2 | Forward | GAAACGCTGAGTCGTTGGTTAGGC | E1 |
| 42 | 3mSbp2 | Reverse | CAGATTTCAAACTGCTCTCAG | E4 |
| 43 | 5hSBP2E1 | Forward | GAAACGCTTTGTCTGTCCGGCAAG | E1 |
| 44 | 3hSBP2E4 | Reverse | CTGATTTCAAACTATTCTCAGCG | E4 |
| 45 | 5′hnRNP H | Forward | ATATAT***ACGCGT***ATGTTGGGCACGGAAGGTGGAG | MluI |
| 46 | 3′hnRNP H | Reverse | ATATA***GTCGAC***CTATGCAATGTTTGATTGAAAA TCACTGG | SalI |
| 47 | F1 | Forward | CGCGGGTTTGCTATGGCGAT | SMN E1 |
| 48 | R1 | Reverse | CAGAATCATCGCT**CTGGCCTGT** | SMN Jxn E1/E2A |
| 49 | F2 | Forward | TTCCTTCTGGACCACCA**ATAA** | SMN Jxn E5/E6 |
| 50 | R2 | Reverse | TCTATGCCAGCATTTC**TCCTTAATTTAAG** | SMN Jxn E7/E8 |
| 51 | F3 | Forward | AGACTGGGACCAGGAAA**GATAA** | SMN Jxn E4/E6 |
| 52 | R3 | Reverse | TGCTCTATGCCAGCATTTC**CATAT** | SMN Jxn E6/E8 |
| 53 | F4 | Forward | TGCAGCTTCCTTACAACAG**AATGA** | SMN Jxn E2B/E4 |
| 54 | R4 | Reverse | CTTTCCTGGTCCCAGTCTTG | SMN E4 |
| 55 | 5′ACTB | Forward | CTCTGGCTCCTAGCACCATGAAGA |  |
| 56 | 3′ACTB | Reverse | GTAAAACGCAGCTCAGTAACAGTCCG |  |
| 57 | 5′HMBS | Forward | GAGTCTAGATGGCTCAGATAGCATGC |  |
| 58 | 3′HMBS | Reverse | CCTACAGACCAGTTAGCGCACATC |  |
| 59 | 5′GAPDH | Forward | GTCTCCTGCGACTTCAACAG |  |
| 60 | 3′GAPDH | Reverse | TCATTGTCATACCGGAAATGAGC |  |
| 61 | 3E8-25 | Reverse | TTAGTGCTGCTCTATGCCAGCATTT | SMN E8 |
| 62 | 5SMNE1TSS | Forward | CGCGGGTTTGCTATGGCGAT | E1 |
| 63 | 5SMNE8-St | Forward | GAAATGCTGGCATAGAGCAGCAC | E8, SalI |
| 64 | 3Adapter-SalI | Reverse | CGAATTCTAGAG***GTCGAC***GCAG |  |
| 65 | 3Adapter-oligo(dT) | Reverse | CGAATTCTAGAGGTCGACGCAGGTTTTTTTTTTTTTTTTTVN |  |
| 66 | 5SMNE1-MluI | Forward | TAGC***ACGCGT***GCGATGAGCAGCGGCGGCAG | E1, MluI |

**Note:** Abbreviations, E, Exon; Jxn, Junction. Exon junction sites are in bold and underline. Enzyme sites are in bold and italic.
