# Supplementary material for: Oxidative Stress Triggers Body-Wide Skipping of Multiple Exons of the Spinal Muscular Atrophy Gene
Source: PLoS One. 2016 Apr 25;11(4):e0154390. doi: 10.1371/journal.pone.0154390 (PMC4844106; doi:10.1371/journal.pone.0154390)
Supplement: S2 Table — (DOCX) [file pone.0154390.s009.docx]

**S2 Table.** A list of altered proteins from PQ treated TG brain

| Protein ID | Mowse score | Mass | pI | Coverage (%) | Change in PQ |
| --- | --- | --- | --- | --- | --- |
| Heterogeneous nuclear ribonucleoprotein H1 | 120 | 49454 | 5.89 | 8 | Down |
| Alpha-enolase 1 | 304 | 47453 | 6.37 | 23 | Down |
| Lactate dehydrogenase B | 83 | 36834 | 5.7 | 7 | Up |
| Phosphoglyceratemutase 1 | 50 | 28928 | 6.67 | 4 | Up |

Mowse: Molecular Weight Search
