# Supplementary material for: Oxidative Stress Triggers Body-Wide Skipping of Multiple Exons of the Spinal Muscular Atrophy Gene
Source: PLoS One. 2016 Apr 25;11(4):e0154390. doi: 10.1371/journal.pone.0154390 (PMC4844106; doi:10.1371/journal.pone.0154390)
Supplement: S3 Table — (DOCX) [file pone.0154390.s010.docx]

**S3 Table.** Description and GenBank accession numbers of mammalian expression vectors

| **Mammalian expression vector** | **GenBank accession number** |
| --- | --- |
| pCI-NEO-FLAG-SMN | KU847909 |
| pCI-NEO-FLAG-SMN Δ5 | KU870437 |
| pCI-NEO-FLAG-SMN Δ7 | KU870438 |
| pCI-NEO-FLAG-SMN Δ5,6 | KU870439 |
| pCI-NEO-FLAG-SMN Δ5,7 | KU870440 |
| pCI-NEO-FLAG-SMN Δ5,6,7 | KU870441 |
| pCI-NEO-FLAG-SMN Δ3 | KU870442 |
| pCI-NEO-FLAG-SMN Δ3,4,5,6,7 | KU870443 |
| pCI-NEO-FLAG-SMN Δ3,5,7 | KU870444 |
| pCI-NEO-FLAG-SMN Δ3,5,6 | KU870445 |
| pCI-NEO-FLAG-SMN Δ3,7 | KU870446 |
| pCI-NEO-FLAG-SMN Δ3,5 | KU870447 |
